# Towards a Comprehensive Characterization of the Low-Temperature Autoxidation of Di-n-Butyl Ether

**DOI:** 10.3390/molecules26237174

**Published:** 2021-11-26

**Authors:** Nesrine Belhadj, Maxence Lailliau, Roland Benoit, Philippe Dagaut

**Affiliations:** 1 Centre National de la Recherche Scientifique–Institut National des Sciences de l’Ingénierie et des Systèmes, Institut de Combustion, Aérothermique, Réactivité, Environnement, 1C Avenue de la Recherche Scientifique, CEDEX 2, 45071 Orléans, France; nesrine.belhadj@cnrs-orleans.fr (N.B.); maxence.lailliau@cnrs-orleans.fr (M.L.); roland.benoit@cnrs-orleans.fr (R.B.); 2 Collegium Sciences et Techniques, Rue de Chartres, Université d’Orléans, 45100 Orléans, France

**Keywords:** di-n-butyl ether, jet-stirred reactor, low-temperature oxidation, atmospheric chemistry, high resolution mass spectrometry, liquid chromatography, kinetic modeling

## Abstract

In the present study, we investigated the oxidation of 2500 ppm of di-n-butyl ether under fuel-rich conditions (φ = 2) at low temperatures (460–780 K), a residence time of 1 s, and 10 atm. The experiments were carried out in a fused silica jet-stirred reactor. Oxidation products were identified and quantified in gas samples by gas chromatography and Fourier transform infrared spectrometry. Samples were also trapped through bubbling in cool acetonitrile for high-pressure liquid chromatography (HPLC) analyses. 2,4-dinitro-phenylhydrazine was used to derivatize carbonyl products and distinguish them from other isomers. HPLC coupled to high resolution mass spectrometry (Orbitrap Q-Exactive^®^) allowed for the detection of oxygenated species never observed before, i.e., low-temperature oxidation products (C_8_H_12_O_4,6_, C_8_H_16_O_3,5,7_, and C_8_H_18_O_2_,_5_) and species that are more specific products of atmospheric oxidation, i.e., C_16_H_34_O_4_, C_11_H_24_O_3_, C_11_H_22_O_3_, and C_10_H_22_O_3_. Flow injection analyses indicated the presence of high molecular weight oxygenated products (*m/z* > 550). These results highlight the strong similitude in terms of classes of oxidation products of combustion and atmospheric oxidation, and through autoxidation processes. A kinetic modeling of the present experiments indicated some discrepancies with the present data.

## 1. Introduction

The use of fossil fuels contributes to anthropogenic emissions of CO_2_ and the formation of particles in the atmosphere, which are harmful to the ecosystem and cause health problems [1]. In addition, the increase in global energy needs and the depletion of fossil resources create socio-economic problems [2] which lead to the exploration of other energy sources such as nuclear, hydro, wind, solar, and renewable fuels from biomass which do not compete with food resources [3]. The chemical composition of a fuel induces specific physico-chemical characteristics, e.g., viscosity, density, boiling point, etc., as well as combustion and oxidation specificities. These characters influence the performance of internal combustion engines and the formation of pollutants and particles [4]. The development of future oxygenated lignocellulosic biofuels has put di-n-butyl ether (DBE) at the ranking of the most promising lignocellulosic biofuel and fuel additive [5]. Various experimental studies have highlighted the combustion characteristics of di-n-butyl ether and its effect on the performance of engines and injection systems [6,7,8], particle emissions [9], soot formation [10,11], and flame characteristics [12]. The combustion of di-n-butyl ether has been explored in different experimental systems and over various temperature and pressure ranges. Fan et al. [13] investigated its pyrolysis kinetics using a flow reactor experiment at low and atmospheric pressures. They identified and quantified several intermediates and products using photoionization molecular-beam mass spectrometry (PI-MBMS). Thion et al. [14] studied the oxidation of 1000 ppm of di-n-butyl ether at low to high temperatures (470 to 1250 K) in a fused silica jet-stirred reactor (JSR) at 1 and 10 atm. Oxidized species have been identified by gas chromatography-mass spectrometry (GC-MS) and Fourier transform infrared spectrometry (FTIR). GC analyses have also been performed using mass spectrometry, flame ionization, and thermal conductivity detectors. The authors observed two negative temperature coefficient regions (NTC). They also proposed a detailed chemical kinetic reaction mechanism to simulate their JSR experiments and model literature data. Tran et al. [15] studied the oxidation of di-n-butyl ether at 400–1100 K and the nearly atmospheric pressure using different experimental systems, i.e., a plug flow reactor (PFR) combined with electron ionization molecular-beam mass spectrometry (EI-MBMS) as well as two different jet-stirred reactors with either online gas chromatography or synchrotron vacuum ultraviolet photoionization molecular-beam mass spectrometry (SVUV-PI-MBMS). They confirmed two negative temperature coefficient zones around 500–550 K and 650–750 K, as reported earlier by Thion et al. [14]. About 40 species have been reported, among which 20 low-temperature products were not previously observed, such as hydroperoxides (CH_4_O_2_, C_3_H_8_O_2_, and C_8_H_18_O_3_), ketohydroperoxides (C_8_H_16_O_4_), and some highly oxygenated molecules (C_8_H_14_O_5_ and C_8_H_16_O_6,8_) [15]. They also proposed a detailed chemical kinetic reaction mechanism to simulate their experiments [15].

More recently, an experimental study has been carried out in our research team [16]. A jet-stirred reactor has been used to study the cool flame oxidation of 5000 ppm of di-n-butyl ether (i.e., 520 K and 480 to 670 K at 1 and 10 atm, respectively). A rapid compression machine (RCM) has also been used to perform ignition experiments of DBE/an air mixture at 550–630 K and 5 bar. High resolution mass spectrometry (HRMS) analyses of JSR and RCM chemical samples have revealed the formation of hydroperoxides (C_8_H_18_O_3_), ketohydroperoxides (C_8_H_16_O_4_), and a range of highly oxygenated molecules (HOMs), namely C_8_H_14_O_5,7_ and C_8_H_16_O_6,8_. However, that study focused on what can be called combustion chemistry, i.e., fuel + X^•^ (^•^OH, H^•^, O^•^, HO_2_^•^, O_2_…) → R^•^ + XH; R^•^ + O_2_ → ROO^•^ → ^•^QOOH (H-shift); ^•^QOOH + O_2_ → ^•^OOQOOH → HOOQ′=O + ^•^OH, and further H-shifts and O_2_ additions. The comparison of the JSR and RCM results indicated strong similitude in terms of low-temperature products detected using these two experimental devices. However, that study, as well as other previous studies, overlooked the formation of other oxygenated products via oxidation channels considered more representative of atmospheric chemistry, i.e., 2 ROO^•^ → ROOOOR → 2 RO^•^ + O_2_; RO^•^→ ^•^QOH (H-shift); ^•^QOH + O_2_ → ^•^OOQOH → OQ’OH + ^•^OH, and further H-shifts and O_2_ additions; ROO^•^+ R’OO^•^ → ROOR’ + O_2_; ROO^•^+ R’OO^•^ → ROH + R’_(‒H)_=O + O_2_. Thus, a more comprehensive study is necessary to clarify the oxidation processes occurring during the cool flame oxidation of di-n-butyl ether and to assess the occurrence of pathways other than extended oxidation routes observed earlier [17,18,19]. Moreover, whereas several studies have shown a similarity between combustion processes and those involved in atmospheric chemistry, this has not been addressed for DBE.

The aim of the present work is to better characterize the low-temperature oxidation chemistry of DBE and to probe the possible formation of products of importance in an atmospheric chemistry such as highly oxidized molecules (HOMs), products of RO^•^ isomerization and oxidation, and accretion products. To this end, the oxidation of 2500 ppm of di-n-butyl ether was performed at low temperature (460–780 K), a residence time of 1 s, and high pressure (10 atm) in a fused silica jet-stirred reactor. Products of DBE oxidation through both ‘combustion’ and ‘atmospheric’ oxidation routes were tracked using gas chromatography, Fourier transform infrared spectrometry, and ultra-high pressure/ high-pressure liquid chromatography (UHPLC/HPLC) coupled to HRMS. An existing kinetic reaction mechanism [14] was used to model the present experiments.

## 2. Results and Discussion

Di-n-butyl ether low-temperature (460–780 K) and high-pressure (10 atm) oxidation leads to the formation of several molecular intermediates and products, including low mass chemical species, i.e., H_2_, CO, CO_2,_ H_2_O, alkanes (methane, ethane, and propane), olefines (ethylene, propene, 1,3-butadiene, and 1-butene), carbonyls (formaldehyde, acetaldehyde, propionaldehyde, butyraldehyde, acetone, acrolein, and 2-butanone), carboxylic acids (formic acid, acetic acid, and butyric acid), alcohols (methanol, ethanol, and 1-butanol), and low mass cyclic ethers (oxirane and 2,3-DHF). All these compounds were identified by FTIR, gas chromatography (GC), or liquid chromatography (LC-HRMS) using standards. Their profiles were plotted and compared to modeling results using a literature kinetic reaction scheme [14] (see Supplementary Materials, Figures S1–S3).

The present study focuses on characterizing low-temperature intermediates of oxidation such as DBE-yl-hydroperoxides (C_8_H_18_O_3,5_), unsaturated DBE-yl-hydroperoxides (C_8_H_16_O_3,5,7_), ketohydroperoxides (C_3_H_6_O_3_, C_4_H_8_O_3_, C_5_H_10_O_4_, C_6_H_12_O_4_, and C_8_H_16_O_4_), diones (C_8_H_14_O_3_), cyclic ethers and butoxy-carbonyls (C_8_H_16_O_2_), and highly oxygenated molecules (C_8_H_12_O_4,6_, C_8_H_14_O_5,7_, and C_8_H_16_O_6,8_).

**Figure 1:**
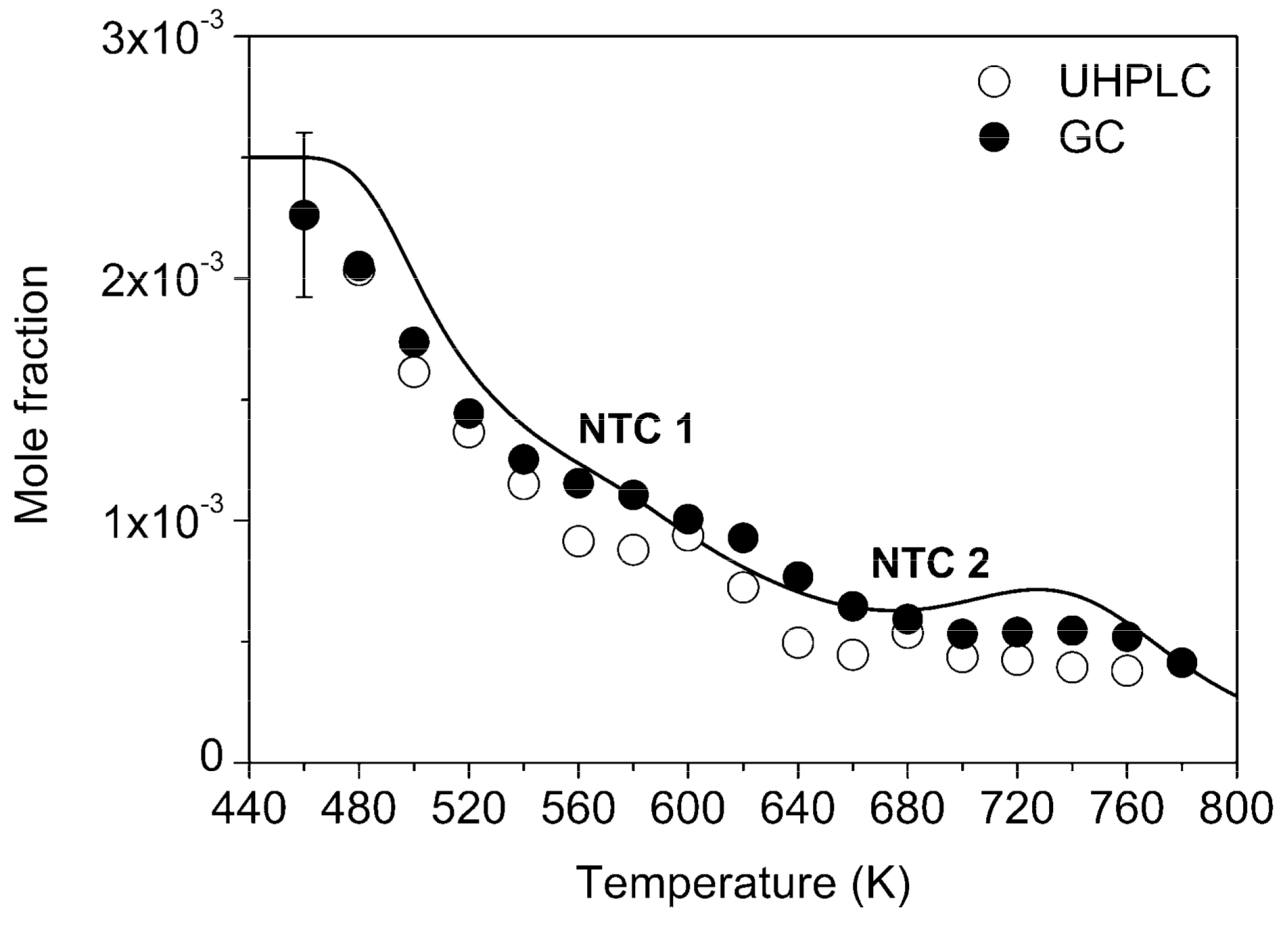
DBE mole fraction profile during its low-temperature (460–780 K) and high-pressure (10 atm) oxidation in a JSR (2500 ppm of DBE, φ = 2, and residence time of 1 s). The line corresponds to modeling results and symbols to both GC and UHPLC experimental results (UHPLC-positive APCI-HRMS, C_8_H_19_O^+^, *m/z* 131.1430). Simulations were performed with the model of Thion et al. [14].

Figure 1 presents the variation of the mole fraction of di-n-butyl ether as a function of temperature. As can be seen from that figure, two NTCs were observed under the present conditions. This is in line with what was reported earlier [14,15]. We observed maximal fuel conversion at ~540 and ~660 K. The kinetic model tends to represent the GC data fairly well, whereas those from Orbitrap analyses were scaled to the GC mole fraction at the lowest oxidation temperature common to the two types of experiments (480 K), i.e., GC, FTIR, and HPLC-MS.

The normalized rate of consumption analysis was performed with PSR [20] at 540 K and 660 K. It indicated that DBE is essentially consumed by H-atom abstraction by hydroxyl radicals at both temperatures. However, the formation of hydroxyl radicals proceeds via different routes when temperature increases from 540 to 660 K. In the first cool flame, the production of hydroxyl radicals is predominantly due to the decomposition of C_8_ ^•^OOQOOH radicals deriving from the fuel (54.3%) and, to some extent, to C_8_ KHPs’ decomposition (29.1%). Minor contributions of the decomposition of C_3_H_7_OOH (1.6%) and C_4_ ^•^OOQOOH (3.7%) were predicted. At 660K, 25% of the hydroxyl radical production was due to the decomposition of C_1_–C_3_ ROOH: C_3_H_7_OOH (12%), C_2_H_5_OOH (7.1%), and CH_3_OOH (5.8%). At 660 K, the decomposition of C_8_ ^•^OOQOOH radicals and C_8_ KHPs represents only 13.1% and 11.7%, respectively. A small contribution to ^•^OH formation also comes from the successive decomposition of C_4_ and C_3_ intermediates: C_4_ ^•^OOQOOH → ^•^OH + KHP (3%) → ^•^OH + carbonyls (2.6%) and C_3_ KHPs → carbonyls + ^•^OH (4.4%). This behavior is responsible for the observed two-stage cool flame oxidation of DBE as initially reported by Thion et al. [14], notably similar to what was reported recently for the cool flame oxidation of di-n-propyl ether in a JSR at 700 Torr [21].

### 2.1. Oxygenated Intermediates Formed in Combustion via the RO_2_^•^ Route

#### 2.1.1. Hydroperoxides (C_8_H_18_O_3_), Dihydroperoxides (C_8_H_18_O_5_), Unsaturated Hydroperoxides (C_8_H_16_O_3_), Unsaturated Dihydroperoxides (C_8_H_16_O_5_), and Unsaturated Trihydroperoxides (C_8_H_16_O_7_)

The peroxidation of the fuel’s radicals (Reaction 1) and H-atom abstraction by RO_2_^•^ (Reactions 2 and 3) can yield hydroperoxides (ROOH), i.e., C_8_H_18_O_3_ in the case of di-n-butyl ether.

R^•^ + O_2_ ⇄ RO_2_^•^, i.e., ^•^C_8_H_17_O + O_2_ ⇄ ^•^OOC_8_H_17_O (1)

RO_2_^•^ + R’H →ROOH + R’^•^, i.e., ^•^OOC_8_H_17_O + R’H → C_8_H_18_O_3_ + R’^•^ (2)

RO_2_^•^ + HO_2_^•^ →ROOH + O_2_, i.e., ^•^OOC_8_H_17_O + HO_2_^•^ → C_8_H_18_O_3_ + O_2_ (3)

**Scheme 1:**
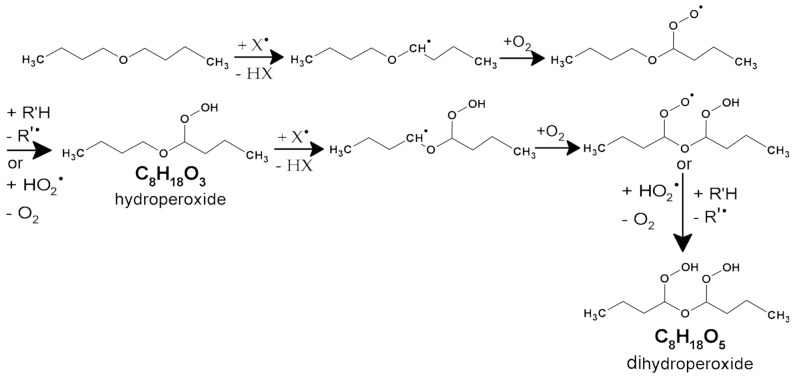
Proposed formation pathways for hydroperoxides (C_8_H_18_O_3_) and dihydroperoxides (C_8_H_18_O_5_) during the low-temperature oxidation of di-n-butyl ether.

Further reactions can yield dihydroperoxides (di-ROOH), i.e., C_8_H_18_O_5_, as shown in Scheme 1.

**Figure 2:**
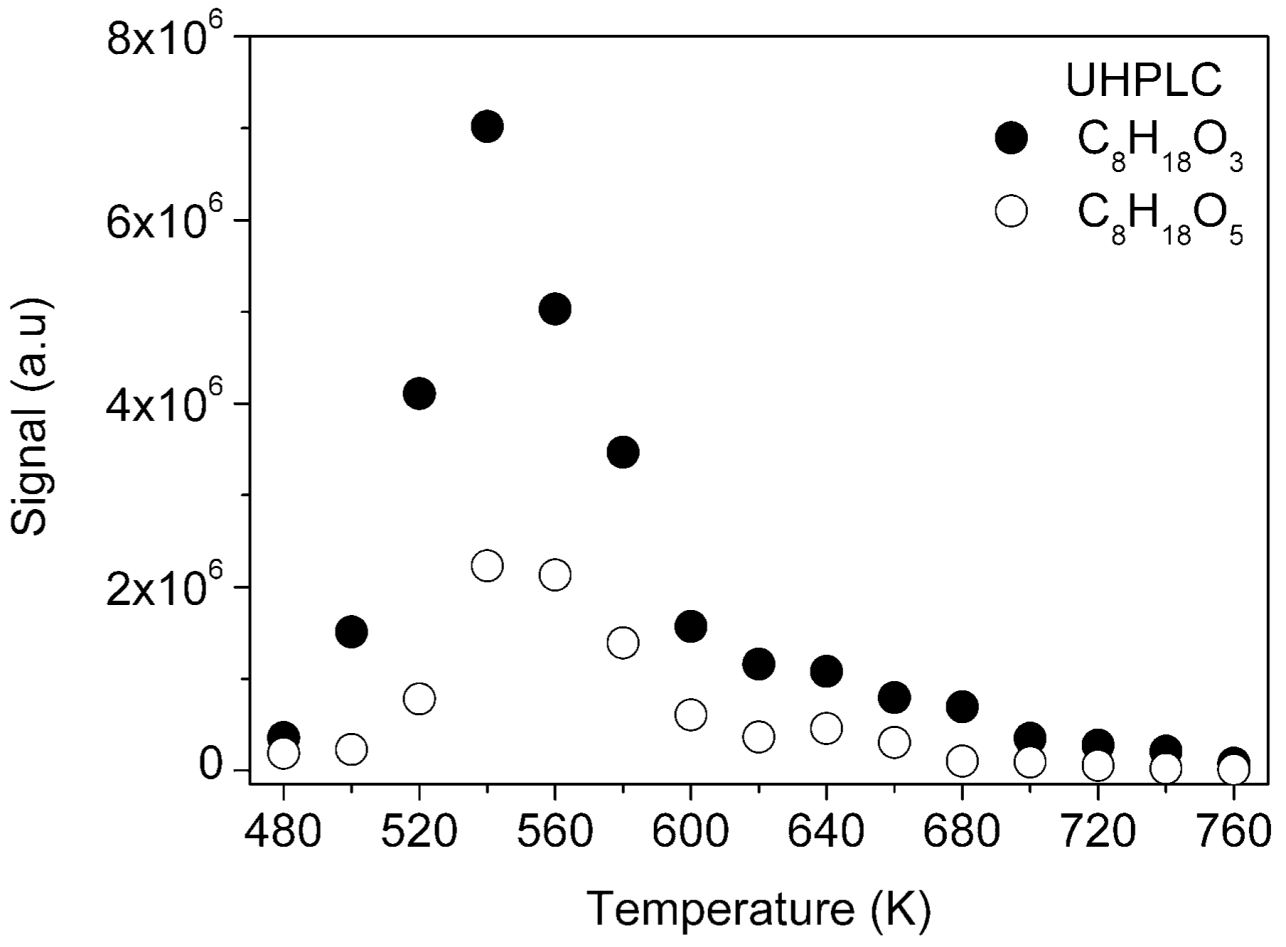
Experimental UHPLC HRMS signal profiles for ROOH, C_8_H_18_O_3_ (positive APCI, C_8_H_19_O_3_^+^), and di-ROOH, C_8_H_18_O_5_ (negative APCI, C_8_H_17_O_5_^−^), formed during the low-temperature oxidation of di-n-butyl ether.

Due to the use of high-resolution mass spectrometry, signals corresponding to C_8_H_18_O_3_ and C_8_H_18_O_5_ were detected using a C_18_ UHPLC column for products’ separation and ionization by positive and negative APCI (C_8_H_19_O_3_^+^, *m/z* 163.1327 and C_8_H_17_O_5_^−^, *m/z* 193.1081). Profiles are plotted in Figure 2. One can note that both C_8_H_18_O_3_ and C_8_H_18_O_5_ hydroperoxides reach a maximum at 540 K, which corresponds to the maximum amplitude of the first DBE cool flame. A second moderate increase of concentration (signal) was observed at ~640 K, which is close to the maximum amplitude of the second DBE cool flame (~660 K).

**Scheme 2:**
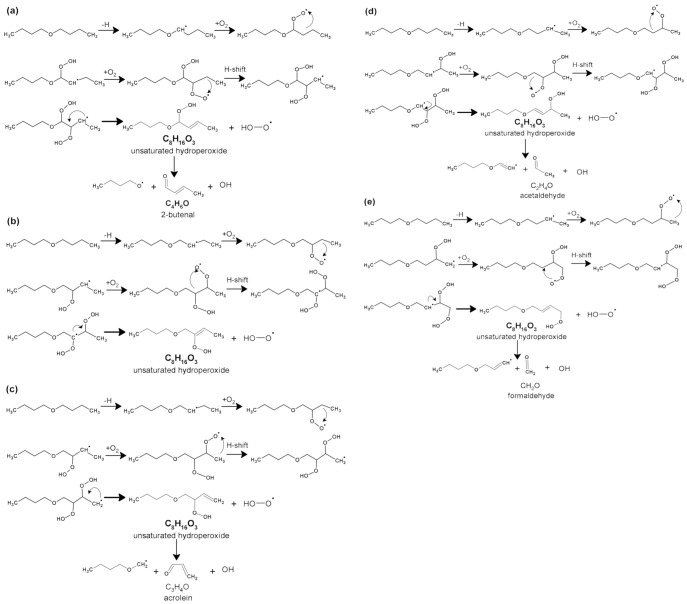
Proposed formation pathways for unsaturated hydroperoxides (C_8_H_16_O_3_) during the low-temperature oxidation of di-n-butyl ether (**a**–**e**).

C_8_H_18_O_3_ hydroperoxides isomers can undergo H-atom abstraction followed by O_2_ addition, H-shift, and loss of a HO_2_ radical to form unsaturated hydroperoxides (C_8_H_16_O_3_; Scheme 2a–e). The degradation of these unsaturated hydroperoxides (C_8_H_16_O_3_) can produce carbonyl species (e.g., 2-butenal, acrolein, acetaldehyde, and formaldehyde). These products were identified here. Mole fraction profiles and simulations for acrolein, acetaldehyde, and formaldehyde are presented in the Supplementary Materials (Figures S2 and S3). Additionally, 2-butenal was identified in di-n-butyl ether oxidation samples through the formation and detection of the DNPH derivative (crotonaldehyde + DNPH) standard.

**Scheme 3:**
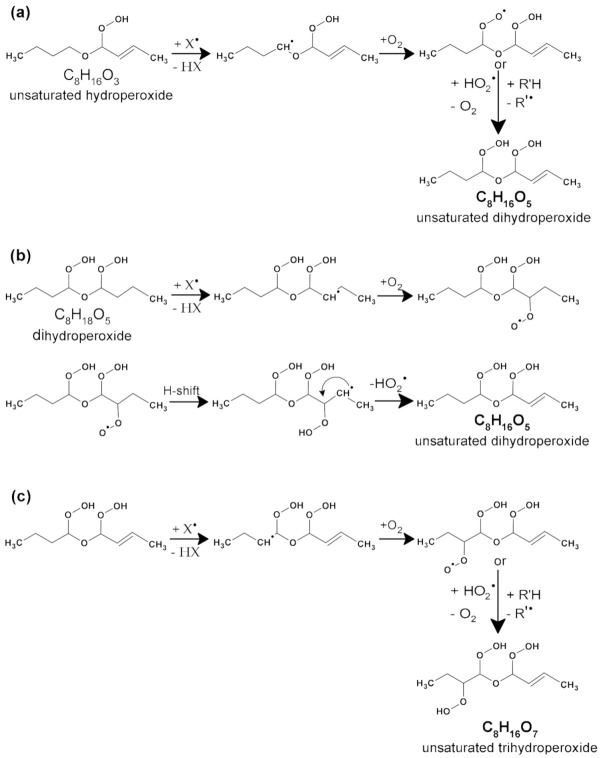
Proposed formation routes for (**a**,**b**) unsaturated di-hydroperoxides (C_8_H_16_O_5_) and (**c**) unsaturated tri-hydroperoxides (C_8_H_16_O_7_) during the low-temperature oxidation of di-n-butyl ether.

Unsaturated di-hydroperoxides (C_8_H_16_O_5_) and unsaturated tri-hydroperoxides (C_8_H_16_O_7_) can also be formed during the low-temperature oxidation of di-n-butyl ether. Different formation routes can be proposed, as illustrated in Scheme 3a–c.

C_18_-UHPLC HRMS analyses revealed the presence of C_8_H_16_O_3_, C_8_H_16_O_5_, and C_8_H_16_O_7_ species, which can correspond to unsaturated hydroperoxides (C_8_H_16_O_3_), unsaturated di-hydroperoxides (C_8_H_16_O_5_), and unsaturated tri-hydroperoxides (C_8_H_16_O_7_). However, these chemical formulae can correspond to other molecules formed via atmospheric oxidation routes. Thus, we will discuss these experimental results later in the article (Section 2.2).

#### 2.1.2. Formation of Ketohydroperoxides (C_3_H_6_O_3_, C_4_H_8_O_3_, C_5_H_10_O_4_, C_6_H_12_O_4_, and C_8_H_16_O_4_), Diketones (C_8_H_14_O_3_), Cyclic Ethers (C_8_H_16_O_2_), and Highly Oxygenated Molecules (C_8_H_12_O_4,6_, C_8_H_14_O_5,7_, and C_8_H_16_O_6,8_)

As mentioned in the introduction, the combustion of fuels is based on the reaction sequences initiated by the abstraction of a hydrogen atom from the fuel. In the case of di-n-butyl ether, the oxidation proceeds via the initial formation of a ^•^C_8_H_17_O radical: C_8_H_18_O + X^•^ (^•^OH, H^•^, O^•^, HO_2_^•^,…) → ^•^C_8_H_17_O + HX, followed by a molecular oxygen addition and H-shift → ^•^OOC_8_H_17_O → HOO^•^C_8_H_16_O, as well as a second O_2_ addition and H-shift → HOOC_8_H_16_O(OO^•^)→ HOO^•^C_8_H_15_O(OOH) which can lose ^•^OH and form O=C_8_H_15_O(OOH) corresponding to keto-hydroperoxides (C_8_H_16_O_4_). If the H-shift involves a non-oxygenated CH group, a third O_2_ addition followed by a H-shift to form (HOO)_2_^•^C_8_H_14_O(OOH) can occur, forming the first highly oxygenated molecule O=C_8_H_14_O(OOH)_2_ known as keto-di-hydroperoxides (C_8_H_16_O_6_), or the process can undergo a fourth O_2_ addition followed by a H-atom transfer to form (HOO)_3_^•^C_8_H_13_O(OOH), which can decompose and yield ^•^OH + keto-tri-hydroperoxides (C_8_H_16_O_8_): (O=)^•^C_8_H_13_O(OOH)_3_. The multi-step autoxidation products (keto-hydroperoxides C_8_H_16_O_4_, keto-dihydroperoxides C_8_H_16_O_6_, and keto-tri-hydroperoxides C_8_H_16_O_8_) can undergo H-abstraction, lose OH from the OOH group, and form species with one or more carbonyl groups [22]:

C_8_H_16_O_4_ (keto-hydroperoxides) + X → HX + OH + C_8_H_14_O_3_ (diketones)
C_8_H_16_O_6_ (keto-di-hydroperoxides) + X → HX + OH + C_8_H_14_O_5_ (di-keto-hydroperoxides)
C_8_H_14_O_5_ (di-keto-hydroperoxides**)** + X → HX + OH + C_8_H_12_O_4_ (tri-ketones)
C_8_H_16_O_8_ (keto-tri-hydroperoxides) + X → HX + OH + C_8_H_14_O_7_ (di-keto-di-hydroperoxides)
C_8_H_14_O_7_ (di-keto-di-hydroperoxides) + X → HX + OH + C_8_H_12_O_6_ (tri-keto-hydroperoxides)

**Scheme 4:**
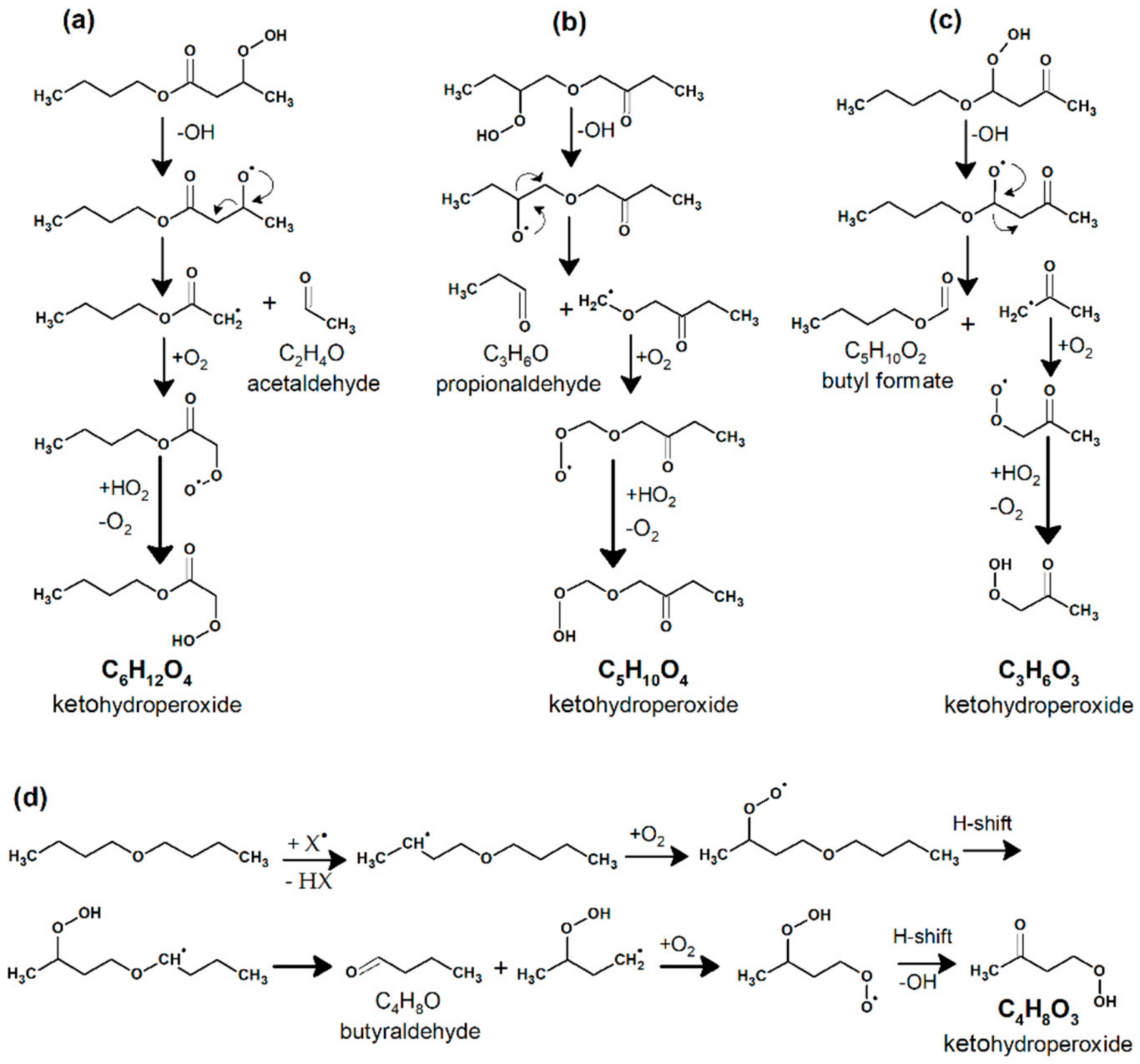
Proposed formation routes for C_3,4,5,6_ ketohydroperoxides (**a**–**d**).

Proposed pathways leading to the formation of C_3_, C_4_, C_5_, and C_6_ KHPs are presented in Scheme 4. These ketohydroperoxides (C_3_H_6_O_3_, C_4_H_8_O_3_, C_5_H_10_O_4_, and C_6_H_12_O_4_) can be formed from different low-temperature oxidation intermediates, i.e., from the decomposition of C_8_-ketohydroperoxides (Scheme 4a–c) and C_8_-peroxy radicals’ reactions (Scheme 4d). Their decomposition produces hydroxyl radicals contributing to the observed second cool flame (620–720 K), as shown in Figure 1.

**Figure 3:**
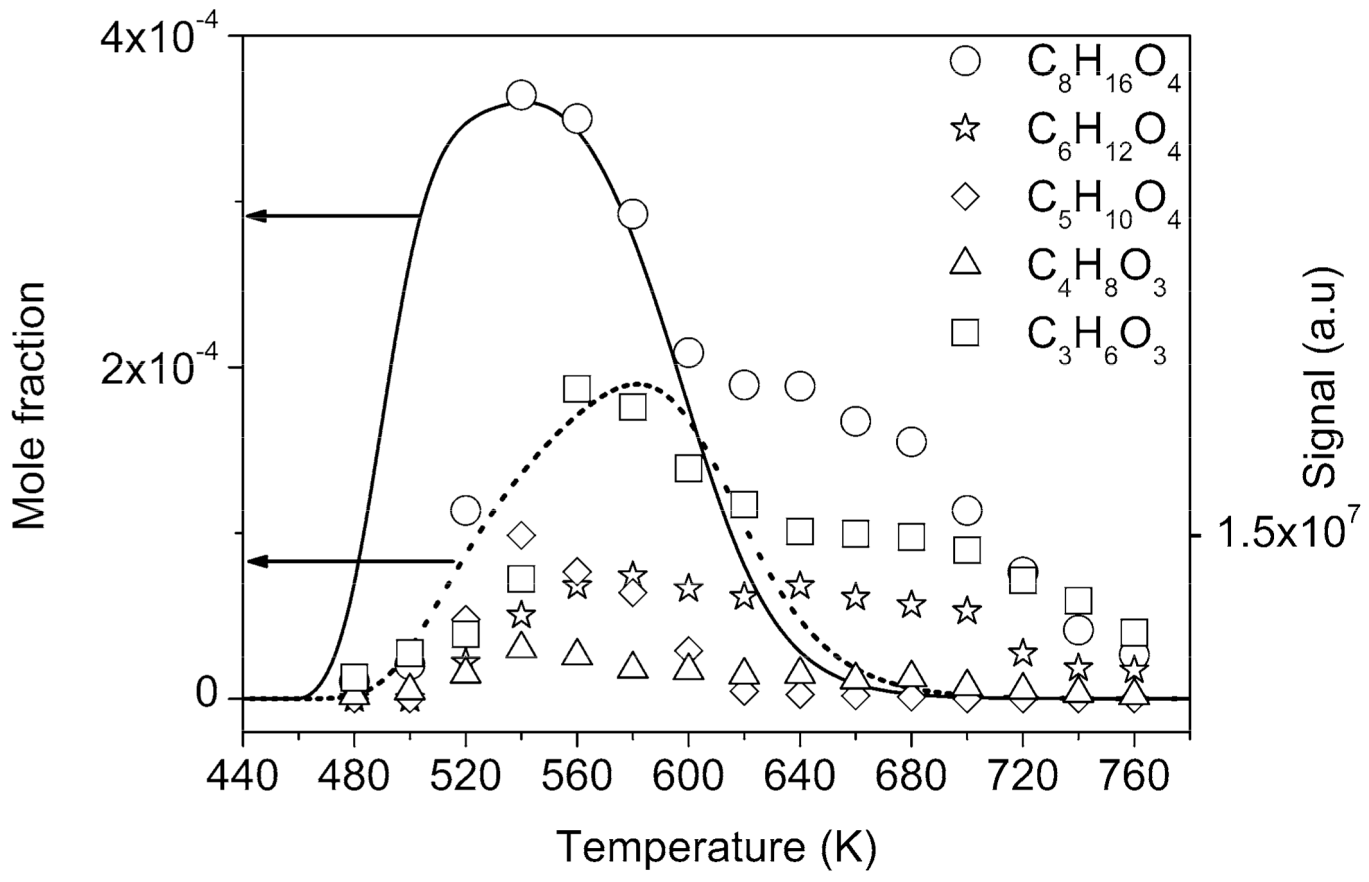
Comparison of the mole fraction profiles for the C_3_H_6_O_3_, C_4_H_8_O_3_, C_5_H_10_O_4_, C_6_H_12_O_4_, and C_8_H_16_O_4_ KHPs formed during the low-temperature oxidation of 2500 ppm of DBE. The continuous line represents the computed C_8_H_16_O_4_ mole fractions, while the dashed line represents the computed C_3_H_6_O_3_ mole fractions. Experimental data are represented by symbols.

Due to the use of liquid chromatography (HPLC and UHPLC) coupled to high resolution mass spectrometry and positive or negative APCI modes, signals corresponding to C_3,4,5,6,8_ ketohydroperoxides C_3_H_6_O_3_ (C_3_H_5_O_3_^−^, *m/z* 89.0243), C_4_H_8_O_3_ (C_4_H_9_O_3_^+^, *m/z* 105.0543), C_5_H_10_O_4_ (C_5_H_11_O_4_^+^, *m/z* 135.0651), C_6_H_12_O_4_ (C_6_H_13_O_4_^+^, *m/z* 149.0807), and C_8_H_16_O_4_ (C_8_H_17_O_4_^+^, *m/z* 177.1121) were detected (chromatograms are given in Supplementary Materials, Figure S4). Their signal profiles were plotted and compared to the modeling results (Figure 3). One should note that only the formation of C_3_H_6_O_3_ and C_8_H_16_O_4_ was been considered in the model of Thion et al. [14]. For convenience, LC-HRMS data for C_3_ and C_8_ KHPs were scaled to their maximum simulated mole fractions. For the other KHPs, we present the variation of the signal of their positive ions as a function of temperature. Considering the low signals obtained for C_5_ and C_6_ KHPs, the data were multiplied by 100. As can be seen from Figure 3, the kinetic model predicts a maximum mole fraction of C_8_H_16_O_4_ at about 540 K, which is in agreement with the experimental data. The model predicts the maximum concentration of C_3_H_6_O_3_ at 580 K, which is close to what was observed experimentally. For the other KHPs, the maximum signal was recorded at ~580 K (C_6_H_12_O_4_) and 540 K (C_5_H_10_O_4_ and C_4_H_8_O_3_).

**Figure 4:**
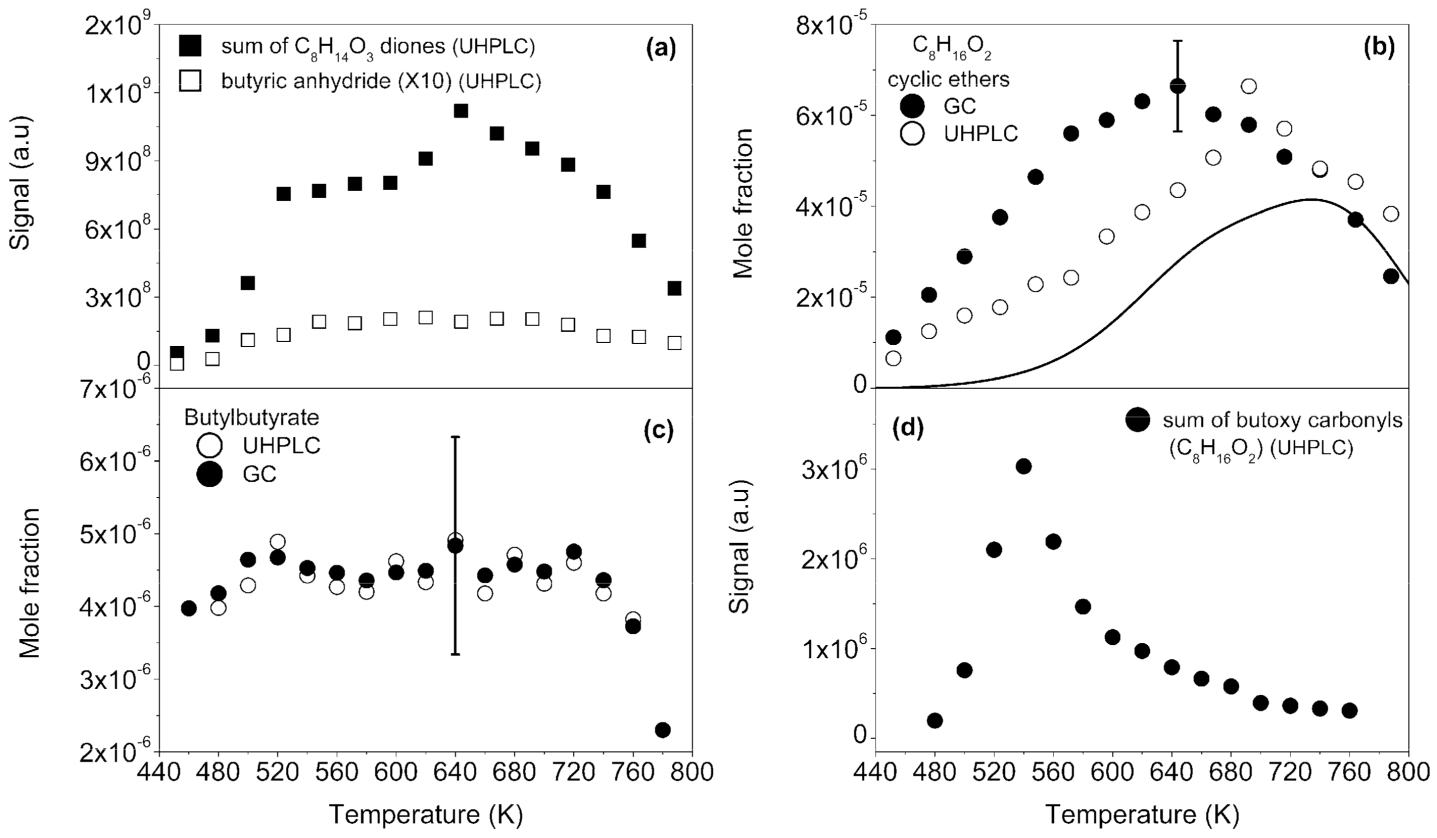
Comparison between mole fractions/signal profiles of diones (C_8_H_14_O_3_), cyclic ethers (C_8_H_16_O_2_), and other C_8_H_16_O_2_ isomers formed during the low-temperature oxidation of di-n-butyl ether. (**a**): Comparison between UHPLC-HRMS experimental results for butyric anhydride and the sum of diones signal. (**b**): Comparison between experimental GC and UHPLC-HRMS data for cyclic ethers (C_8_H_16_O_2_) and simulations (line). (**c**): Comparison between GC and UHPLC-HRMS experimental mole fractions of butyl butyrate (C_8_H_16_O_2_). (**d**) UHPLC-HRMS-positive APCI signal profile of R’_(‒H)_=O species corresponding to C_8_H_16_O_2_ butoxy-carbonyl isomers.

As previously shown [22], diones (here, di-keto-ethers) can be produced from ketohydroperoxides, i.e., C_8_H_16_O_4_ (keto-hydroperoxides) + X → HX + OH + C_8_H_14_O_3_ (diketones). A signal corresponding to di-keto-ethers (C_8_H_14_O_3_) was detected by C_18_-UHPLC HRMS as well as positive APCI (C_8_H_15_O_3_^+^, *m/z* 159.1015). The chromatograms revealed the presence of several C_8_H_14_O_3_ isomers (found in the Supplementary Materials, Figure S5, presenting a C_8_H_15_O_3_^+^ UHPLC-HRMS chromatogram). Only butyric anhydride was identified. The unavailability of diketo-ethers standards made it difficult to identify them. The UHPLC-HRMS experimental profiles for butyric anhydride and the sum of the other diones are presented in Figure 4a.

Cyclic ethers (C_8_H_16_O_2_) can be formed during di-n-butyl ether oxidation [14,15]. They are produced by the decomposition of QO_2_H radicals: ^•^QOOH → cyclic ethers + ^•^OH, although in the case of DBE, one can write HOO^•^C_8_H_16_O → C_8_H_16_O_2_ + ^•^OH. These cyclic ethers have the same molecular formula as esters or keto-ethers (C_8_H_16_O_2_), such as butyl butyrate (n-butyl butanoate), 4-butoxybutanal, 4-butoxybutan-2-one, and 1-butoxybutan-2-one. n-Butyl butanoate can be formed from DBE-yl-hydroperoxides (C_8_H_18_O_3_) and DBE-yl-hydroperoxy radicals (C_8_H_17_O_3_^•^) via H-abstractions from ROOH or via the recombination/disproportionation of RO_2_^•^ [15]. 4-Butoxybutanal can be formed through ROO^•^ self-reaction: ROO^•^+ R’OO^•^ → ROH + R’_(‒H)_=O + O_2_ (cf. Reaction 12 in Section 2.2). Unfortunately, the unavailability of 4-butoxybutanal, 4-butoxybutan-2-one, and 1-butoxybutan-2-one standards made their identification impossible.

C_8_H_16_O_2_ species formed during the low-temperature oxidation of di-n-butyl ether were detected through C_18_-UHPLC-positive APCI HRMS analyses. The chromatograms revealed the presence of several C_8_H_16_O_2_ isomers (C_8_H_17_O_2_^+^, *m/z* 145.1222; see Supplementary Materials, Figure S5). 2,4-DNPH derivatization was applied to distinguish cyclic ethers and butoxy-carbonyl C_8_H_16_O_2_ isomers (4-butoxybutanal, 4-butoxybutan-2-one, and 1-butoxybutan-2-one). Cyclic ethers do not react with 2,4-DNPH, whereas carbonyls do. Therefore, the intensity of their chromatographic peaks decreases. Computed and experimental (GC, UHPLC) mole fraction profiles of the sum of cyclic ethers and n-butyl butanoate, and the sum of butoxy-carbonyls isomers are presented in Figure 4b–d.

One should note that unlike the modeling and UHPLC-HRMS data, which reveal the formation of several cyclic ethers (ten species in the kinetic model, i.e., 4-ethyl-2-propyl-1,3-dioxolane, 4-methyl-2-propyl-1,3-dioxane, 2-butoxytetrahydrofuran, etc., and at least five in the UHPLC-HRMS analyses), GC analyses allowed for the detection of only two cyclic ethers. Their maxima of concentration are not reached at the same temperature (640 K in GC, 680 K in UHPLC, and 720 K in the simulation), which could be explained by the absence of the detection of some cyclic ethers in the GC analyses. The kinetic model underestimates the formation of cyclic ethers by about 50%.

Concerning butyl butyrate profiles (Figure 4c), we noticed a very good qualitative agreement between the GC and UHPLC results. In addition, it is noticeable that butyl butyrate reached maxima at 520 K and 640 K. This could be due to its formation via different intermediate species.

Figure 4d shows that the butoxy-carbonyl profile (sum of 4-butoxybutanal, 4-butoxybutan-2-one, and 1-butoxybutan-2-one) peaks at 540 K.

**Scheme 5:**
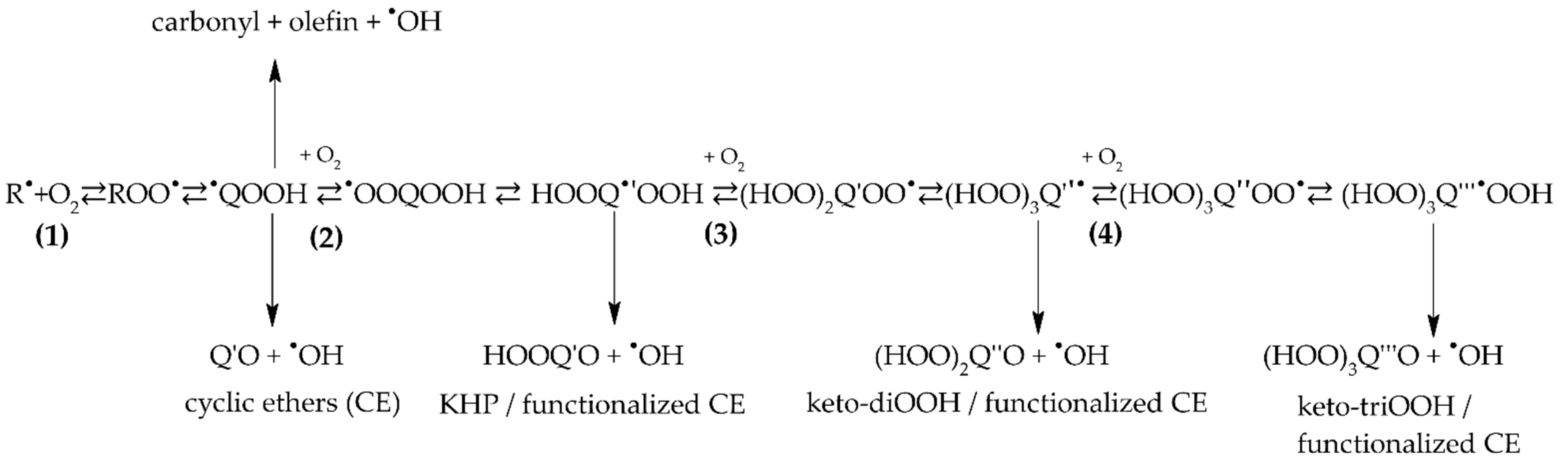
Schematic representation of the low-temperature oxidation pathways including the formation of KHPs, di and tri-hydroperoxides, and functionalized cyclic ethers isomers. (1) to (4) refer to first to fourth O_2_ addition.

One should note that KHPs are isomeric forms of hydroperoxyl-cyclic ethers. We have considered that the chemical formulae detected in this work for these isomers correspond mostly to KHPs. This assumption is based on results of simulations performed for n-hexane under conditions close to those of the present study [23] using a literature mechanism [18], including the formation of functionalized cyclic ethers (hydroperoxy and di-hydroperoxy-cyclic ethers). The model predicted 16 and 160 times less hydroperoxy and di-hydroperoxy-cyclic ethers, respectively, than KHPs. This result seems to validate our assumption. Scheme 5 gives a simplified view of the reaction pathways forming these products.

**Figure 5:**
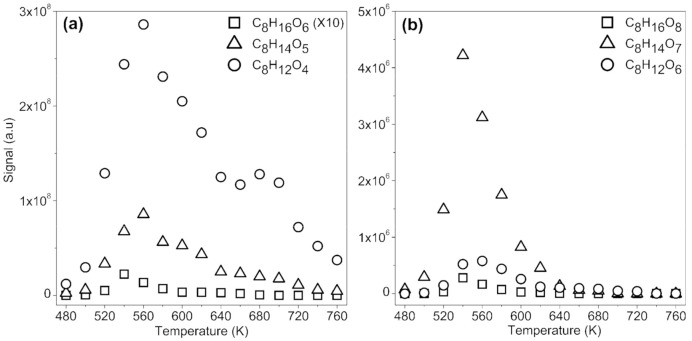
Experimental UHPLC-HRMS signal profiles for multi-oxygenated molecules formed during the low-temperature oxidation of di-n-butyl ether: (**a**) C_8_H_16_O_6_ (negative APCI, C_8_H_15_O_6_^−^, *m/z* 207.0873), C_8_H_14_O_5_ (negative APCI, C_8_H_13_O_5_^−^, *m/z* 189.0768), and C_8_H_12_O_4_ (negative APCI, C_8_H_11_O_4_^−^, *m/z* 171.0662); and (**b**) C_8_H_16_O_8_ (negative APCI, C_8_H_15_O_8_^−^, *m/z* 239.0771), C_8_H_14_O_7_ (positive APCI, C_8_H_15_O_7_^+^, *m/z* 223.0812), and C_8_H_12_O_6_ (positive APCI, C_8_H_13_O_6_^+^, *m/z* 205.0706).

As presented earlier [16] highly oxygenated molecules (C_8_H_16_O_6_ and C_8_H_16_O_8_) resulting from a third and fourth O_2_ addition on fuel radicals were detected using flow injection (FIA-HRMS) analyses. However, in the present study, we developed a C_18_-UHPLC HRMS analytical method. Chromatographic analyses revealed the presence of several C_8_H_16_O_6_ and C_8_H_16_O_8_ peaks (see Supplementary Materials, Figure S6). In addition, the other multi-oxygenated intermediates (C_8_H_12_O_4,6_ and C_8_H_14_O_5,7_), resulting from the decomposition of C_8_H_16_O_6_, and C_8_H_16_O_8_, were also detected using UHPLC HRMS with positive and negative APCI. C_8_H_14_O_5_ (DBE-di-ketohydroperoxides) was observed by Tran et al. [15] and in our earlier study [16], and C_8_H_14_O_7_ (DBE- di-keto-dihydroperoxides) was also observed in our previous study [16], but C_8_H_12_O_4,6_ has never been observed previously. Figure 5a shows UHPLC-HRMS experimental profiles of DBE-keto-dihydroperoxides (C_8_H_16_O_6_), DBE-diketo-hydroperoxides (C_8_H_14_O_5_), and DBE-triketones (C_8_H_12_O_4_). Figure 5b shows UHPLC-HRMS experimental profiles of DBE-keto-trihydroperoxides (C_8_H_16_O_8_), DBE-di-keto-dihydroperoxides (C_8_H_14_O_7_), and DBE-tri-keto-hydroperoxides (C_8_H_12_O_6_). The C_8_H_16_O_6_, C_8_H_14_O_5_, C_8_H_12_O_4_, and C_8_H_16_O_8_ experimental results were obtained using negative APCI. Since these species are not efficiently ionized in the same APCI mode, the C_8_H_12_O_6_ and C_8_H_14_O_7_ data were obtained with positive APCI.

### 2.2. Oxygenated Products Formed via Atmospheric Oxidation Routes (RO_2_^•^ and RO^•^)

Additional oxidation channels not taken into account in the combustion were considered in the atmospheric chemistry, apart from the usual oxidation routes (Reactions (4) and (5)).

R + X^•^
→ R^•^ + XH (4)

R^•^ + O_2_
→ ROO^•^ (5)

Among them, one can find reactions which could also occur during combustion but are generally ignored (Reactions (7)–(12)):

2 ROO^•^
→ ROOOOR → 2 RO^•^ + O_2_ (6)

RO^•^
→ ^•^QOH (H-shift) (7)

^•^QOH + O_2_ → ^•^OOQOH → OQ’OH + ^•^OH (8)

^•^OOQOH → HOO^•^Q’OH (H-shift) (9)

HOO^•^Q’OH + O_2_→ ^•^OO(HOO)Q’OH → O(HOO)Q’’OH + ^•^OH (10)

ROO^•^ +R’OO^•^
→ ROOR’ + O_2_ (11)

ROO^•^ + R’OO^•^ → ROH + R’_(‒H)_=O + O_2_ (12)

Besides, ROH can also be formed through H-atom abstraction by an alkoxy radical:

RO^•^ + XH → ROH + X^•^ (13)

Through Reactions (4)–(10), peroxy radicals (ROO^•^ with R = C_8_H_17_O) can form RO^•^ radicals (here, ^•^OC_8_H_17_O), which undergo successive reactions: H-shift isomerization to form HO^•^C_8_H_16_O followed by O_2_ addition and H-shift yielding (HOO)(HO)^•^C_8_H_15_O. That radical can lose OH by O-O scission in OOH to form C_8_H_16_O_3_ corresponding to O=C_8_H_15_O(HO). Alternatively, it can undergo O_2_ addition and H-atom transfer to form (HOO)_2_(HO)^•^C_8_H_14_O. That radical can either lose an OH radical and form C_8_H_16_O_5_ corresponding to (HOO)(HO)(O=)C_8_H_14_O or undergo O_2_ addition and an H-shift to give (HOO)_3_(HO)^•^C_8_H_13_O, which can lose ^•^OH and form C_8_H_16_O_7_ corresponding to (HOO)_2_(HO)(O=)C_8_H_13_O. One should note that oxygenated molecules (C_8_H_16_O_3,5,7_) formed through atmospheric chemistry pathways present the same chemical formulas as some molecules formed in the fuel’s combustion mechanism. These include C_8_H_16_O_3_, unsaturated DBE-yl-hydroperoxides (Scheme 2a–e), C_8_H_16_O_5_, unsaturated DBE-yl-di-hydroperoxides, and C_8_H_16_O_7_, which can be unsaturated DBE-yl-tri-hydroperoxides (Scheme 3a–c). In order to distinguish these two chemical groups, 2,4-DNPH derivatization was performed (see details in Section 3.2). RP-C_18_ UHPLC-HRMS analyses showed the presence of C_8_H_16_O_3,5,7-_dinitrophenylhydrazone derivatives (C_14_H_20_N_4_O_6,8,10_, respectively). In addition, chromatograms of the C_8_H_16_O_3,5,7_ species obtained before and after DNPH derivatization revealed a decrease of the intensity of some chromatographic peaks, which indicates the presence of carbonyl compounds. The other peaks showing no decreased intensity should be attributed to species having no carbonyl function, e.g., unsaturated hydroperoxides. The chromatograms are given in the Supplementary Materials, Figure S7.

**Figure 6:**
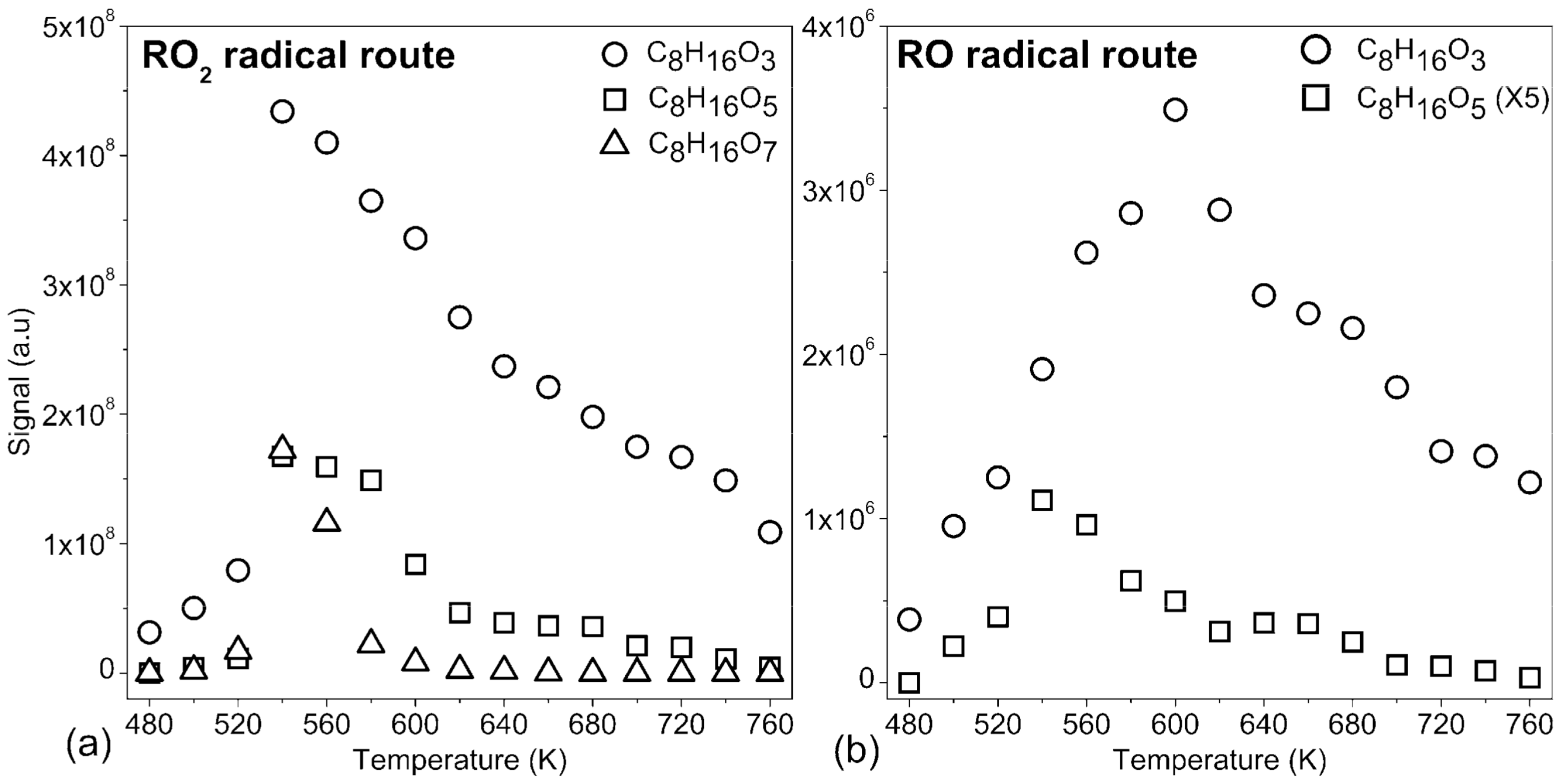
Experimental UHPLC-HRMS signal profiles of C_8_H_16_O_3,5,7_ formed during the low-temperature oxidation of DBE. (**a**) Species formed in combustion: unsaturated DBE-yl-hydroperoxides (C_8_H_16_O_3_), unsaturated DBE-yl-di-hydroperoxides (C_8_H_16_O_5_), and unsaturated DBE-yl-tri-hydroperoxides (C_8_H_16_O_7_) and carbonyls, i.e., (HOO)_2_(HO)(O=)C_8_H_13_O. (**b**) Species formed in the atmospheric RO^•^ oxidation route: O=C_8_H_15_O(HO) (C_8_H_16_O_3_) and (HOO)(HO)(O=)C_8_H_14_O (C_8_H_16_O_5_).

The UHPLC-HRMS analyses of C_8_H_16_O_3,5,7_ (without DNPH derivatization) species were performed using APCI (+/−) modes. However, in Figure 6, only the strongest signals are presented for C_8_H_16_O_3,5_ (APCI+, C_8_H_17_O_3_^+^, *m/z* 161.1172, and C_8_H_17_O_5_^+^, *m/z* 193.1070) and C_8_H_16_O_7_ (APCI‒, C_8_H_15_O_7_^−^, *m/z* 223.0823) isomers. One should note that C_8_H_16_O_7_ isomers were ionized only in the negative APCI mode. Figure 6 shows UHPLC-HRMS signal profiles of C_8_H_16_O_3,5,7_ species formed in combustion reactions. Based on chromatographic analyses with and without the addition of 2,4-DNPH (see Supplementary Materials, Figure S7), the reported data for C_8_H_16_O_7_ (Figure 6a) correspond to different co-eluted compounds, at least one of them containing a carbonyl function which reacted with 2,4-DNPH. UHPLC-HRMS data for C_8_H_16_O_3,5_ are presented in Figure 6b. These products can be formed via atmospheric oxidation routes starting with the isomerization of the initial RO^•^ radical into ^•^QOH (H-shift), followed by an O_2_ addition (Reactions (7)–(10)), i.e., RO^•^→ ^•^QOH (H-shift), ^•^QOH + O_2_ → ^•^OOQOH → OQ’OH + ^•^OH, ^•^OOQOH → HOO^•^QOH (H-shift), and HOO^•^QOH + O_2_→ ^•^OO(HOO)QOH → O(HOO)Q’’OH + ^•^OH).

Let us now focus on the species formed through Reactions (11)–(13), yielding ROOR’, ROH, and R’_(-H)_=O + ROH, respectively.

**Figure 7:**
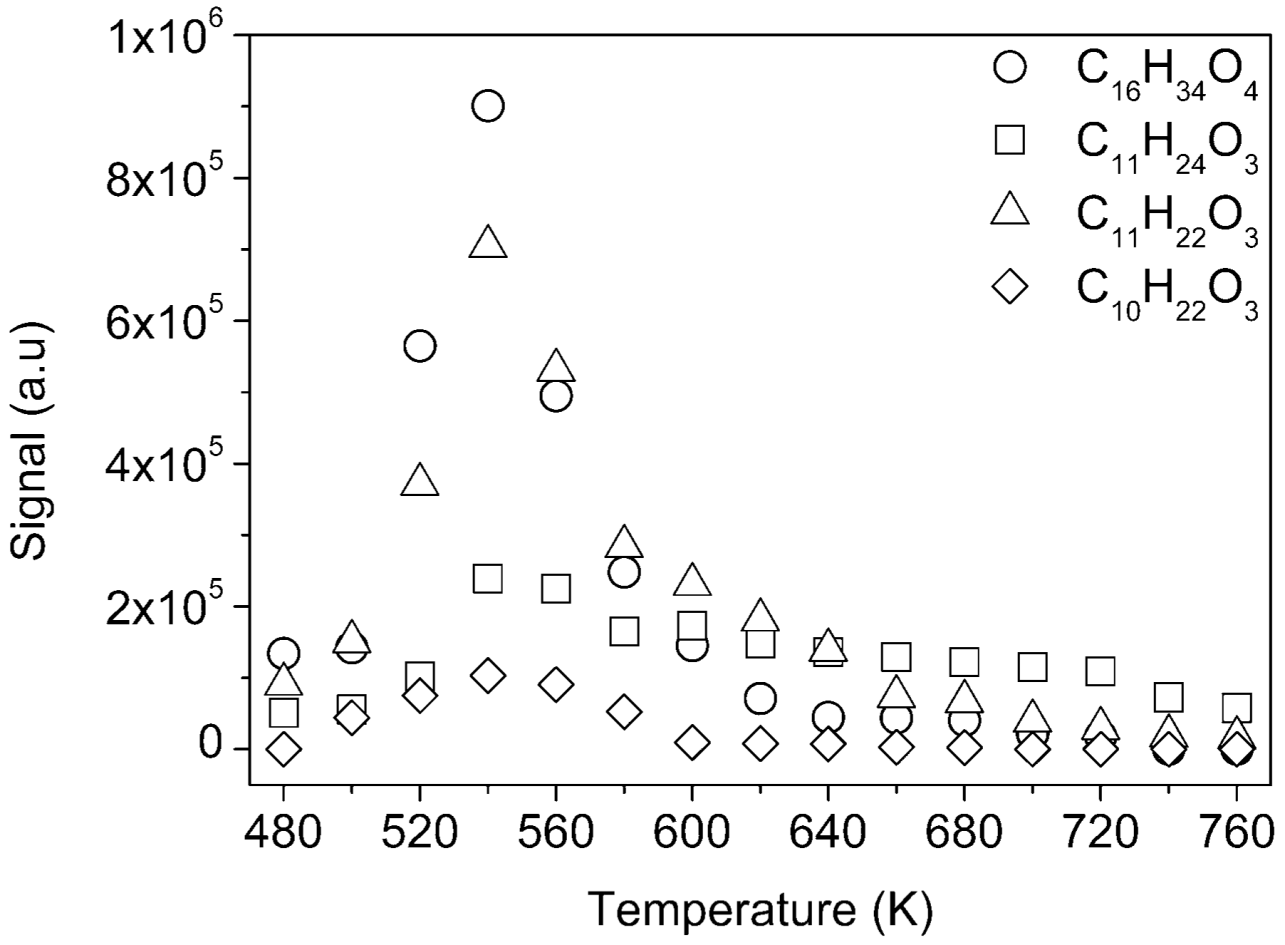
Experimental signal for ROOR’ species formed during the low-temperature oxidation of di-n-butyl ether: C_16_H_34_O_4_ (C_16_H_35_O_4_^+^, *m/z* 291.2528), C_11_H_24_O_3_ (C_11_H_25_O_3_^+^, *m/z* 205.1797), C_11_H_22_O_3_ (C_11_H_23_O_3_^+^, *m/z* 191.1641), and C_10_H_22_O_3_ (C_10_H_23_O_3_^+^, *m/z* 203.1640). The analyses were performed using UHPLC HRMS and positive APCI.

UHPLC-HRMS analyses with positive APCI ionization allowed for the detection of molecules corresponding to ROOR’ species. Among them, one could detect C_16_H_34_O_4_ (with R=R’=C_8_H_17_O), C_11_H_24_O_3_ (with R=C_8_H_17_O and R’=C_3_H_7_), C_11_H_22_O_3_ (with R=C_8_H_15_O and R’=C_3_H_7_), and C_10_H_22_O_3_ (with R=C_8_H_17_O and R’=C_2_H_5_). The evolution of ROOR’ experimental profiles are given in Figure 7. One can see that all ROOR’ (C_16_H_34_O_4_, C_11_H_24_O_3_, C_11_H_22_O_3_, and C_10_H_22_O_3_) reach their maximum intensity at 540 K, which corresponds to the maximum intensity of the first DBE cool flame. The relative importance of their peak intensity varies as follows: C_16_H_34_O_4_ > C_11_H_22_O_3_ > C_11_H_24_O_3_ > C_10_H_22_O_3_.

**Figure 8:**
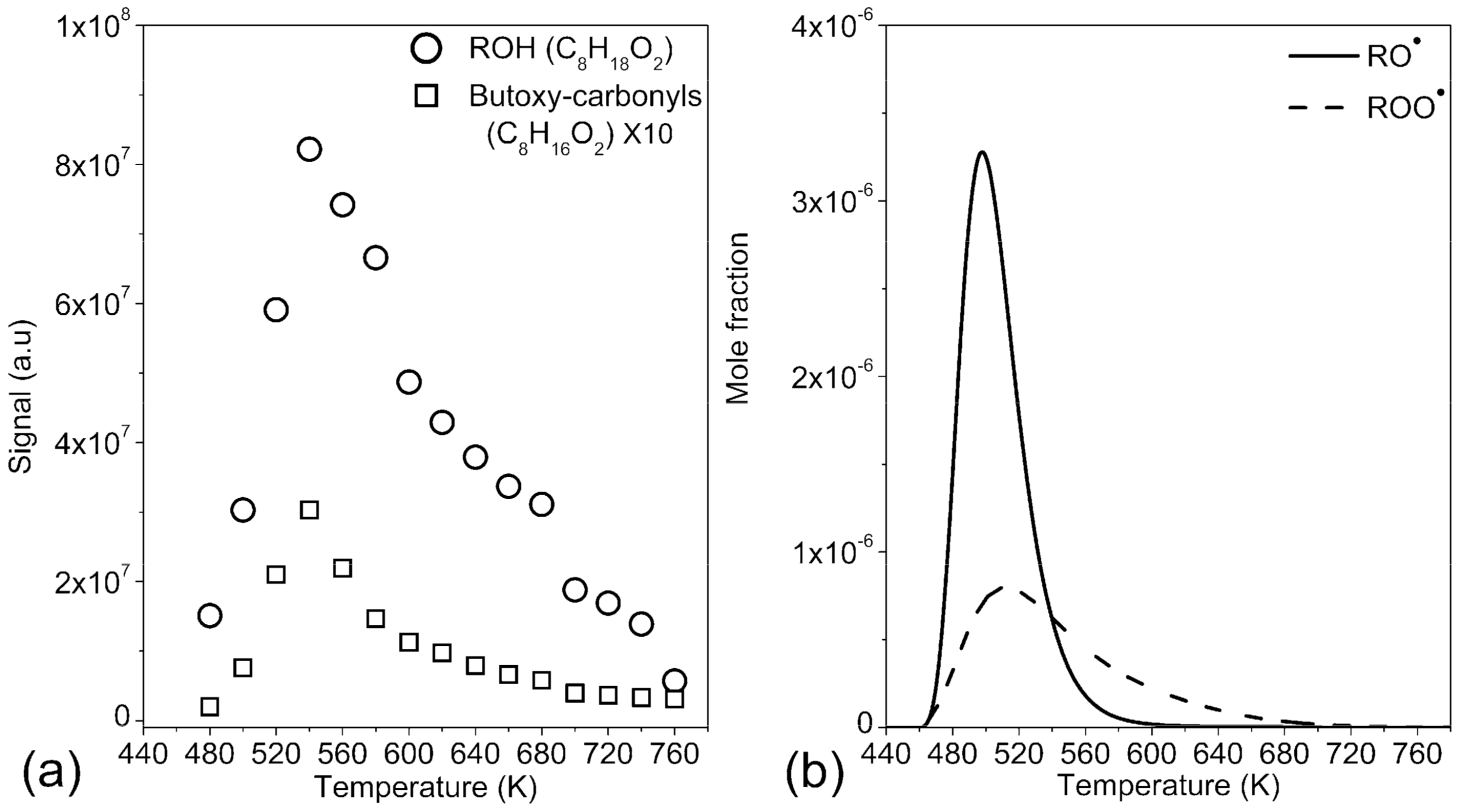
(**a**) Experimental profile of ROH (C_8_H_18_O_2_) and the sum of R’_(‒H)_=O (butoxy-ethers) formed during the low-temperature oxidation of di-n-butyl ether. The analyses were performed using UHPLC HRMS and positive APCI. (**b**) Simulated RO^•^ and ROO^•^ radical mole fractions vs. temperature using the model of Thion et al. [14].

Both Reactions (12) and (13) can form ROH molecules. In the case of di-n-butyl ether, butoxy-butanol species (C_8_H_18_O_2_) were detected using UHPLC HRMS analyses (positive APCI, C_8_H_19_O_2_^+^, *m/z* 147.1378; Figure 8a). We can note that the C_8_H_18_O_2_ signal reached a maximum at 540 K. Based on simulated RO^•^ and ROO^•^ mole fractions (Figure 8b) showing RO^•^ radicals dominating, one can expect that the production of ROH is mainly due to H-atom abstraction reactions by RO^•^ radicals (Reaction (13)). The formation of ROH and R’_‒H_=O, involving ROO^•^ radicals, should be a minor channel under the present conditions as it seems to be based on the observed minor formation of butoxy-carbonyls (Figure 8a).

Concerning the formation of an aldehyde R’_(‒H)_=O via Reaction 12, producing 4-butoxybutanal (C_8_H_16_O_2_), DNPH derivatization was used to distinguish between C_8_H_16_O_2_ isomers (cyclic ethers and carbonyl-ethers). Experimental UHPLC-HRMS results are presented in Section 2.1.2 (Figure 4d) and Section 2.2 (Figure 8a).

### 2.3. Further Characterization of Oxygenated Products

**Figure 9:**
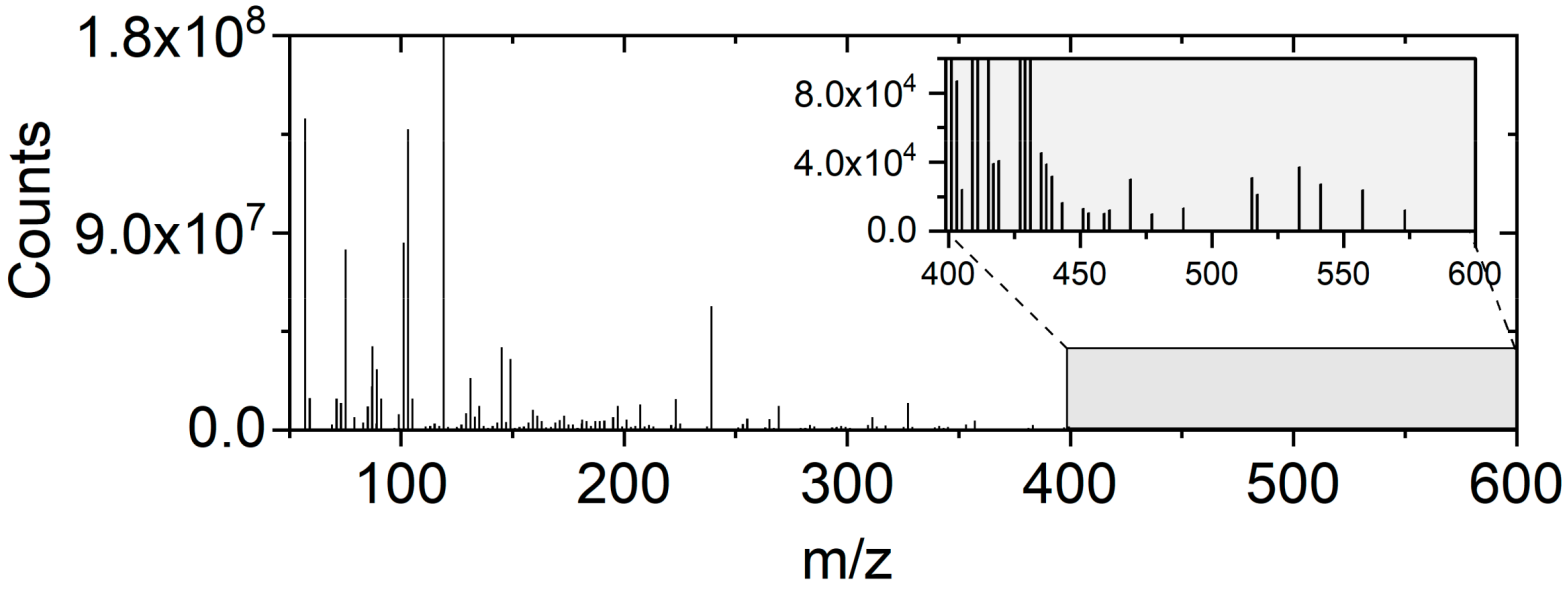
FIA-APCI(‒)-HRMS data obtained with a DBE oxidation sample collected at 540 K.

To further characterize the products of the oxidation of DBE, additional analyses and data processing were performed. A DBE oxidation sample collected at 540 K was analyzed using the flow injection analysis technique (FIA). The analyses were performed using the negative APCI-HRMS parameters given in Table S1 of the Supplementary Materials. As can be seen from the mass spectrum shown in Figure 9, the mass range extended to *m*/*z* > 550. This observation is somewhat unexpected for the oxidation of DBE in a cool flame, whereas it would likely be less surprising if DBE had been oxidized under simulated atmospheric conditions where accretion products have been reported for a range of organics [24].

**Figure 10:**
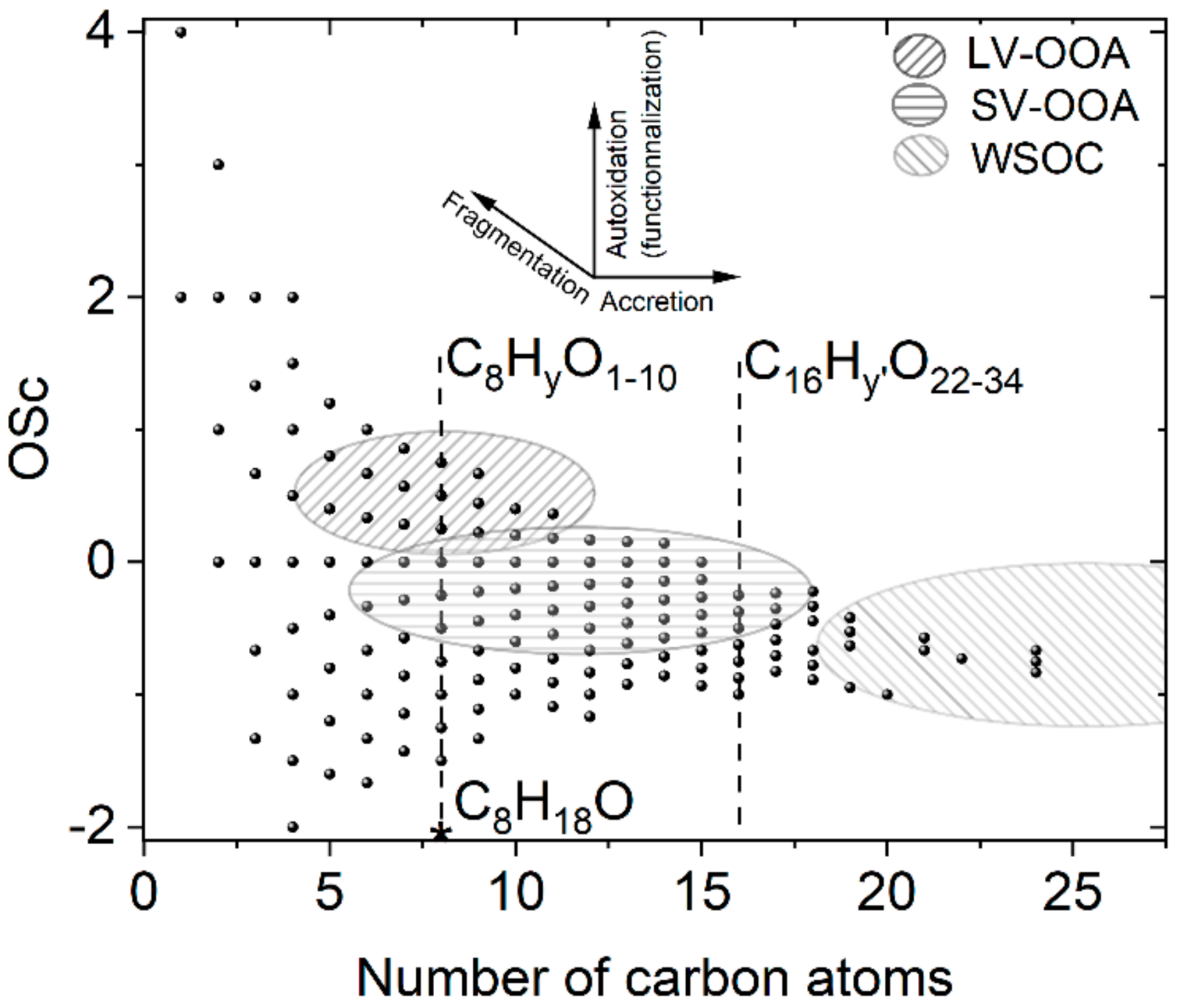
Variation of the OSc as a function of the number of carbon atoms in molecular formulae detected by negative APCI FIA-HRMS of a DBE oxidation sample collected at 540 K.

Unfortunately, no data are available for the atmospheric oxidation of DBE to be compared to the present results. However, in a previous study of ours concerning limonene oxidation in a JSR at atmospheric pressure and 590 K [25], we showed that products of combustion and atmospheric oxidation presented strong similitude in terms of the chemical formulae detected and the variation of the oxidation state of carbon atoms as a function of the number of carbon atoms in the detected formulae. The oxidation state of carbon atoms (OSc ≈ 2O/C–H/C) allows for evaluating the degree of oxidation of a large range of organic species (alcohols, carbonyls, carboxylic acids, esters, and ethers, but not peroxides) [26]. We computed the variation of OSc as a function of the number of carbon atoms, namely nC, for presently detected molecular formulae obtained by HRMS (Figure 10).

One can clearly distinguish several regions corresponding to low-volatility oxygenated organic aerosols (LV-OOA), semi-volatile oxygenated organic aerosols (SV-OOA), and water-soluble organic aerosols (WSOC), as defined by Kroll et al. [26]. Since di-n-butyl ether is composed of eight carbon atoms (C_8_H_18_O), for nC < 8, the observed products result from oxidative fragmentation. For 8 < nC < 16, we observed products of addition or condensation, and for nC > 16, we observed products of oligomerization. A comparison of Figure 10 with results published earlier for the oxidation of limonene in a JSR and with a range of atmospheric oxidation studies [25] indicates unexpected similar trends regarding the presence of LV-OOA, SV-OOA, and WSOC for the two fuels, namely DBE and limonene (see Supplementary Material, Figure S8).

## 3. Materials and Methods

### 3.1. JSR Experiments

The oxidation of DBE was performed in a jet-stirred reactor, which has 4 injectors (nozzles of 1 mm I.D.) providing stirring [27,28,29]. Di-n-butyl ether (>99% purity, Sigma-Aldrich^®^, Saint-Louis, MO, USA) was atomized by a nitrogen (N_2_) gas flow and vaporized in a heated chamber. Diluted oxygen and a fuel–nitrogen mixture were sent separately to the injectors to avoid oxidation before reaching the JSR. The liquid fuel was pumped using a Shimadzu^®^ LC10 AD-VP HPLC pump equipped with a Shimadzu^®^ DGU-20A3 online degasser. N_2_ and O_2_ flow rates were controlled by mass flow controllers (Brooks Instruments, Hatfield, PA, USA,). A Pt-Pt/Rh—10% (0.1 mm O.D., located inside a thin wall jacket) was used to verify good thermal homogeneity (gradients of <1 K/cm) along the vertical axis of the JSR. The experiments were performed twice. First, we collected gas samples for online FTIR and gas phase analysis (GC). Secondly, we collected samples by bubbling gas from the sampling sonic probe into cooled acetonitrile (0 °C). The liquid samples were stored in a freezer (−15 °C) and analyzed by both HPLC and UHPLC coupled to high resolution mass spectrometry (Orbitrap Q-Exactive^®^, Thermo Fisher Scientific, Waltham, MA, USA). This analytical protocol has been used in previous works of ours where its effectiveness was demonstrated [16,23]. The JSR experimental conditions are shown in Table 1. Excellent stability was observed for the fuel, oxygen, and nitrogen flowrates, temperature, and pressure in the JSR during the experiments.

### 3.2. Analytical Procedures

Different complementary analytical methods were developed to analyze di-n-butyl ether oxidation products. Fourier transform infrared spectroscopy was used to detect formic and acetic acids, namely H_2_O, CO_2_, CO, and CH_2_O. Gas chromatography (GC) with a flame ionization detector (FID) to quantify hydrocarbons and oxygenates up to C_8_ was employed. A GC with a thermal conductivity detector (TCD) was employed to quantify H_2_ and O_2_, and a GC-quadrupole mass spectrometer (q-MS) was used for ≤C_8_ products’ identification.

Liquid samples were analyzed by high-pressure and ultra-high-pressure liquid chromatography (HPLC/UHPLC) coupled to an Orbitrap Q-Exactive^®^ mass spectrometer, which has very high resolution (140,000), high mass accuracy (<1 ppm), and a large *m/z* range (50–1800). However, due to the limitation of this mass spectrometer to detect molecules with *m/z* < 50, e.g., CO_2_, CH_4_, C_2_H_4_, C_3_H_6,8_, C_2_H_4_O, CH_2_O, C_2_H_4_O, etc., these molecules were characterized either by FTIR or GC, or after the chemical derivatization of carbonyls with 2,4-dinitrophenyl hydrazine (moistened with water; >99% purity from Sigma-Aldrich^®^) Then, *m/z* increased and derivation products could be detected (carbonyl + 2,4-DNPH = H_2_O + 2,4-DNPHydrazone). To this end, 50 μL of 2,4-DNPH saturated solution (dissolution of 2 g in 100 mL of ACN) and 20 μL of diluted phosphoric acid from Sigma-Aldrich^®^ (100 μL of H_3_PO_4_ in 1 mL of ACN) were added to 1 mL of di-n-butyl ether oxidation samples. The 2,4-DNPH derivatization reaction was carried out at ambient temperatures for 2 h.

Liquid chromatography analyses were mostly performed in the reversed-phase (RP-C_18_ Luna Omega column, Phenomenex^®^, Torrance, CA, USA). Additional high-performance liquid chromatography (HPLC)−HRMS analyses were performed with a Supelco^®^ Ascentis Silica column. To this end, a Vanquish™ system from Thermo Fisher Scientific^®^, Waltham, MA, USA was used. Soft ionization using atmospheric pressure chemical ionization (APCI) in positive ([M+H] ^+^) and negative ([M-H]^−^) modes was used to ionize products after chromatographic separation. Direct infusion was also used (see Supplementary Materials, Table S1). Then, the sample was injected into the ionization chamber of the mass spectrometer using a syringe pump. FTIR, GC, and HPLC/UHPLC analytical settings are detailed in the Supplementary Materials, Table S1. The HPLC-Orbitrap data are only qualitative because no calibration could be done in this work. The other data obtained by GC and FTIR are quantitative because the chemical species (reactants and products) were calibrated using standards. Uncertainties on the mole fractions are of the order of 10% in GC and 15% in FTIR. HOMs are minor products (in the ppm-range); the carbon balance could not be impacted by the fact they are not quantified in this work.

### 3.3. Kinetic Modeling

The present experiments were simulated using the PSR computer code [20]. The detailed kinetic reaction mechanism of Thion et al. [14] was used. It included 2768 reactions (most of them reversible) and 467 chemical species describing both low and high-temperature oxidation of DBE. This mechanism is limited to two additions of O_2_ to fuel radicals (^•^C_8_H_17_O), which yield ketohydroperoxides (C_8_H_16_O_4_).

## 4. Conclusions

The low-temperature (460–780 K) and high-pressure (10 atm) oxidation of 2500 ppm of di-n-butyl ether was studied in a jet-stirred reactor. Due to the use of high-resolution mass spectrometry (Orbitrap Q-Exactive) coupled to liquid chromatography (HPLC and UHPLC) and atmospheric pressure chemical ionization (APCI), a wide range of previously observed and newly observed low-temperature oxidation products were detected. They include, besides light ubiquitous hydrocarbons’ oxidation products, species resulting from the one to four O_2_ additions on the fuel’s radicals, i.e., hydroperoxides and di-hydroperoxides (C_8_H_18_O_3–5_), unsaturated hydroperoxides, di and tri-hydroperoxides (C_8_H_16_O_3,5,7_), ketohydroperoxides (C_3_H_6_O_3_, C_4_H_8_O_3_, C_5_H_10_O_4_, C_6_H_12_O_4_, and C_8_H_16_O_4_), and highly oxygenated molecules (C_8_H_16_O_6,8_, C_8_H_14_O_5,7_, and C_8_H_12_O_4,6_). Oxidation products more specific to atmospheric oxidation chemistry, i.e., C_8_H_16_O_3,5,7_ with carbonyl and hydroxyl groups, and one or more hydroperoxyl groups, i.e., C_8_H_18_O_2_ corresponding to butoxy-butanol species, and ROOR’ compounds, including C_16_H_34_O_4_, C_11_H_24_O_3_, C_11_H_22_O_3_, and C_10_H_22_O_3_, were also detected. The present results allowed for highlighting the finding that common reaction pathways pertaining to combustion and atmospheric autoxidation chemistry occur under the present JSR experimental conditions. Further studies involving complementary techniques, e.g., SVUV-PI-MBMS, would be useful for confirming the proposed identification of isomeric products such as cyclic ethers vs. carbonyls. FIA indicated the formation of high molecular weight oxygenated products (*m*/*z* > 550) under the present experimental conditions. Accretion products are of high importance for the formation of atmospheric particulates. Then, it would be interesting to investigate the formation of the newly detected species in this study using a smog chamber or flow reactors to further clarify similarities in terms of oxidation pathways in combustion and atmospheric oxidation conditions.

## Figures and Tables

**Table 1:** Experimental conditions for the DBE oxidation in a JSR.

Parameters	Values
Reactive mixture	0.25% of DBE, 1.5% of O_2_, 98.25% of N_2_
Equivalence ratio (φ)	2
Residence time (s)	1
Pressure (atm)	10
Temperature (K)	460–780
Volume of acetonitrile (mL)	20
Bubbling time (min)	60

## Data Availability

Data are available from the corresponding author.

## Supplementary Materials

Figure S1: Evolution of O_2_, H_2_O, CO, CO_2_, and H_2_, and formic acid experimental and computed mole fraction profiles obtained for the low-temperature oxidation of 2500 ppm of di-n-butyl ether at 10 atm, φ = 2, and a residence time of 1 s; Figure S2: Comparison between experimental mole fractions of organic species and simulations for the oxidation of 2500 ppm of di-n-butyl ether in JSR (pressure of 10 atm, low-temperature 460–780 K, φ = 2, and a residence time of 1 s); Figure S3: Mole fraction profiles of aldehydes (formaldehyde, acetaldehyde, propionaldehyde, and butyraldehyde), ketones (acetone and 2-butanone), carboxylic acids (acetic acid and butyric acid), and 2,3-DHF formed during the low-temperature oxidation of 2500 ppm of di-n-butyl ether at 10 atm; Figure S4: Chromatograms showing C_3,4,5,6,8_ ketohydroperoxides formed during the oxidation of 2500 ppm of di-n-butyl ether in a JSR (pressure: 10 atm; temperature: 460–780 K; φ = 2; and residence time = 1 s); Figure S5: Top: chromatogram showing the formation of C_8_H_14_O_3_ diones during the oxidation of di-n-butyl ether. Butyric anhydride was identified at 7.18 min. Analyses were performed using a C18 UHPLC column with positive APCI HRMS (C_8_H_15_O_3_^+^ with *m/z* 159.1015). Bottom: chromatogram showing the formation of cyclic ethers, butyl butyrate, and butoxy-carbonyls (C_8_H_16_O_2_) during the oxidation of di-n-butyl ether; Figure S6: Chromatograms showing HOMs formed during the low-temperature oxidation of 2500 ppm of di-n-butyl ether at 10 atm; Figure S7: Chromatograms showing C_8_H_16_O_3,5,7_ obtained before and after DNPH derivatization; Figure S8: Variation of the OSc as a function of the number of carbon atoms in molecular formulae detected by the negative FIA-HRMS of a limonene oxidation sample collected at 590 K and 1 atm; and Table S1: Conditions for GC, normal, and reverse-phase liquid chromatography and FTIR analyses.
