# Supplementary material for: Towards a Comprehensive Characterization of the Low-Temperature Autoxidation of Di-n-Butyl Ether
Source: Molecules. 2021 Nov 26;26(23):7174. doi: 10.3390/molecules26237174 (PMC8658975; doi:10.3390/molecules26237174)
Supplement: Supplementary file 1 [file molecules-26-07174-s001.zip › molecules-1473575-supplementary.pdf]

Supplementary Materials

Towards a comprehensive characterization of the low-temperature autoxidation of di-n-butyl ether

Nesrine Belhadj [1,2], Maxence Lailliau [1,2], Roland Benoit [1], and Philippe Dagaut [1,*]

[1] CNRS–INSIS, ICARE, 1C avenue de la Recherche Scientifique, 45071 Orléans cedex 2, France ;
nesrine.belhadj@cnrs-orleans.fr ; maxence.lailliau@cnrs-orleans.fr ; roland.benoit@cnrs-orleans.fr ; dagaut@cnrs-orleans.fr

[2] Université d'Orléans, rue de Chartres, 45100 Orléans, France ; nesrine.belhadj@etu.univ-orleans.fr

*Correspondence: dagaut@cnrs-orleans.fr

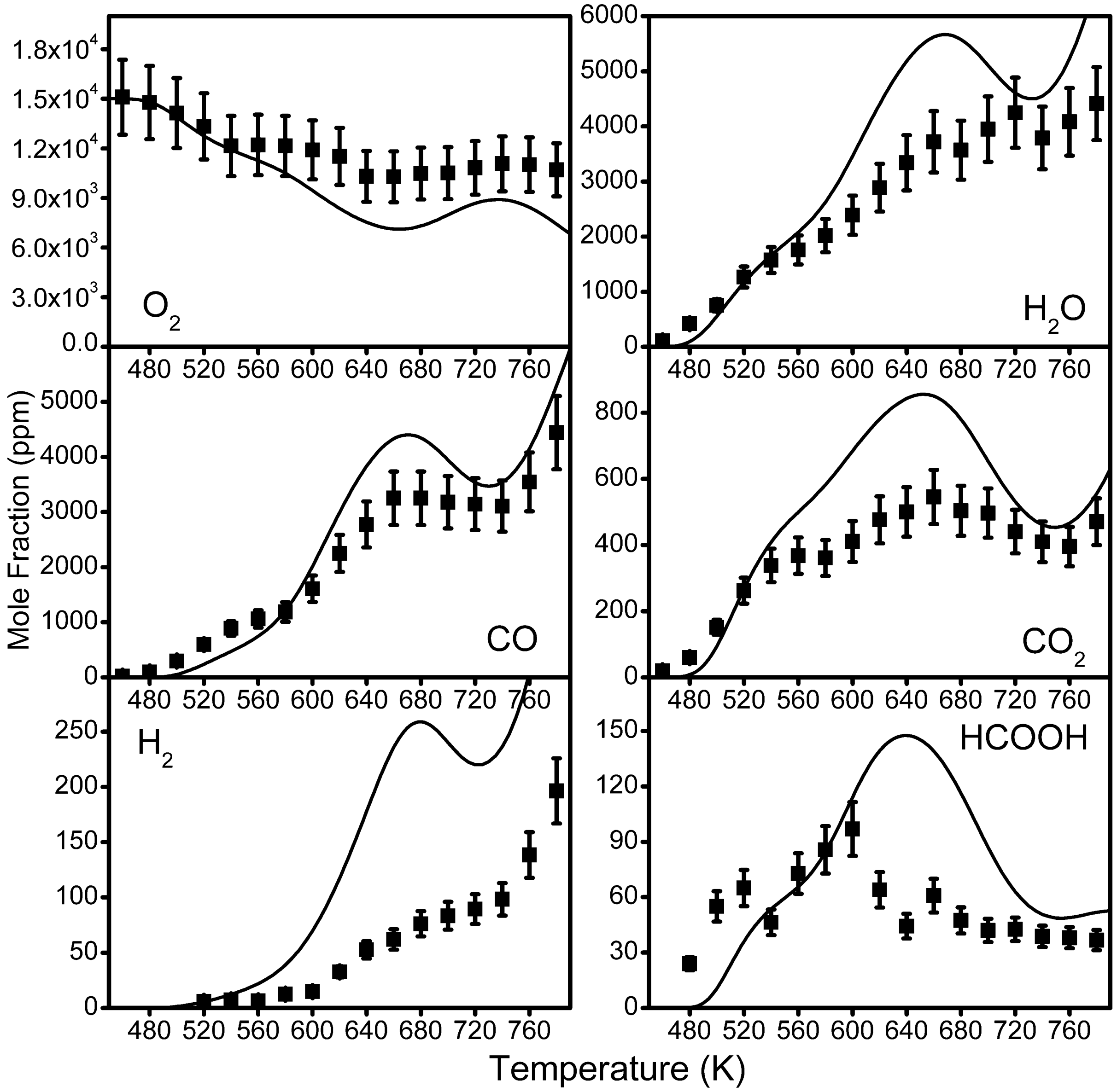

Figure S1: Experimental (symbols) and computed (lines) mole fractions obtained for the low-temperature oxidation of 2500 ppm of di-n-butyl ether at 10 atm, φ = 2 and a residence time of 1 s. Experimental results (symbols) were obtained by GC-TCD (O_2 and H_2), FTIR (CO_2, CO, H_2O, and formic acid).

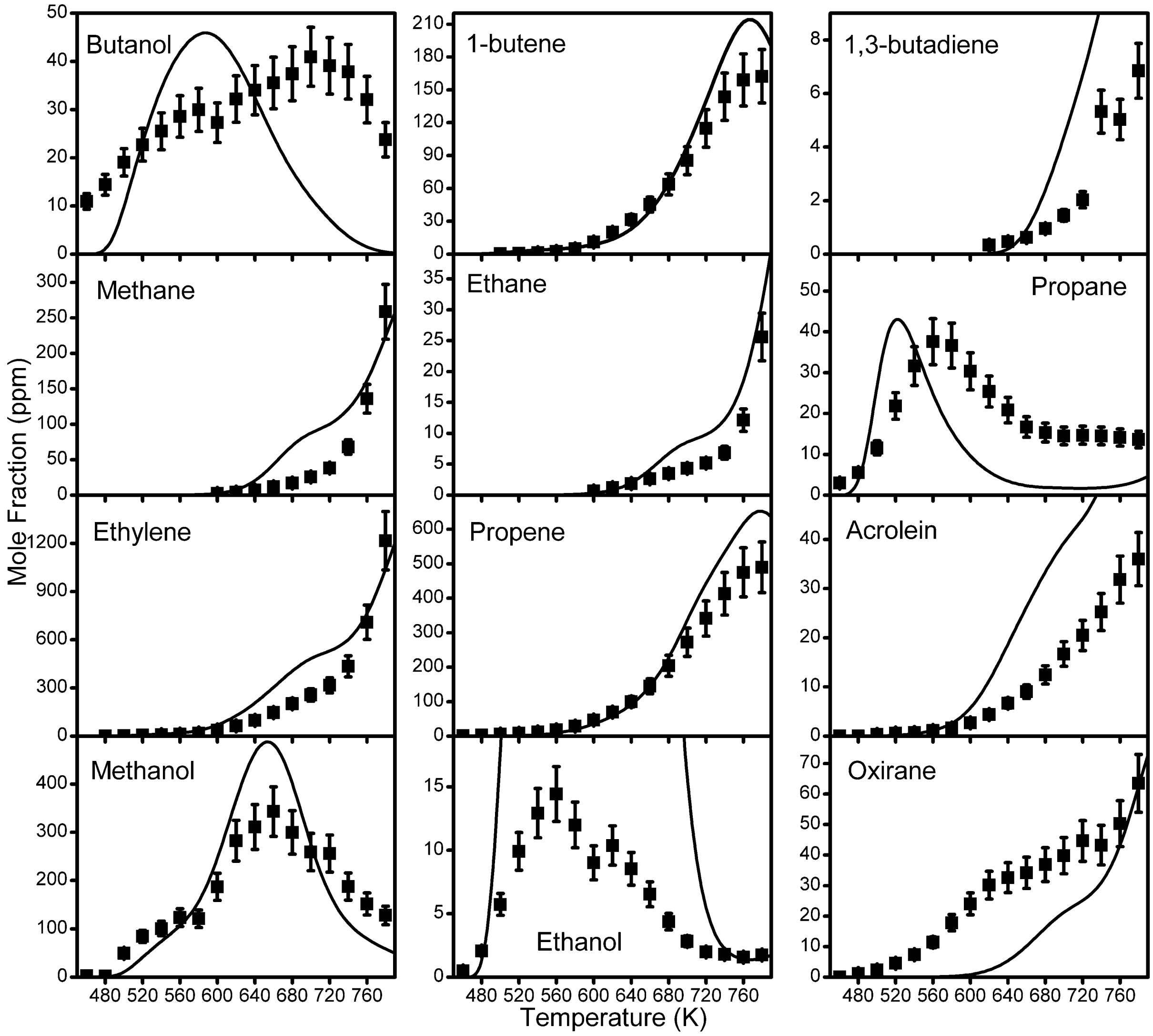

Figure S2: Comparison between experimental mole fractions of organic species and simulations for the oxidation of 2500 ppm of di-n-butyl ether in JSR (pressure of 10 atm, low-temperature 460-780K, φ = 2 and residence time of 1 s). Experimental results (square symbols) were obtained using GC-q-MS; they are compared to simulations (lines).

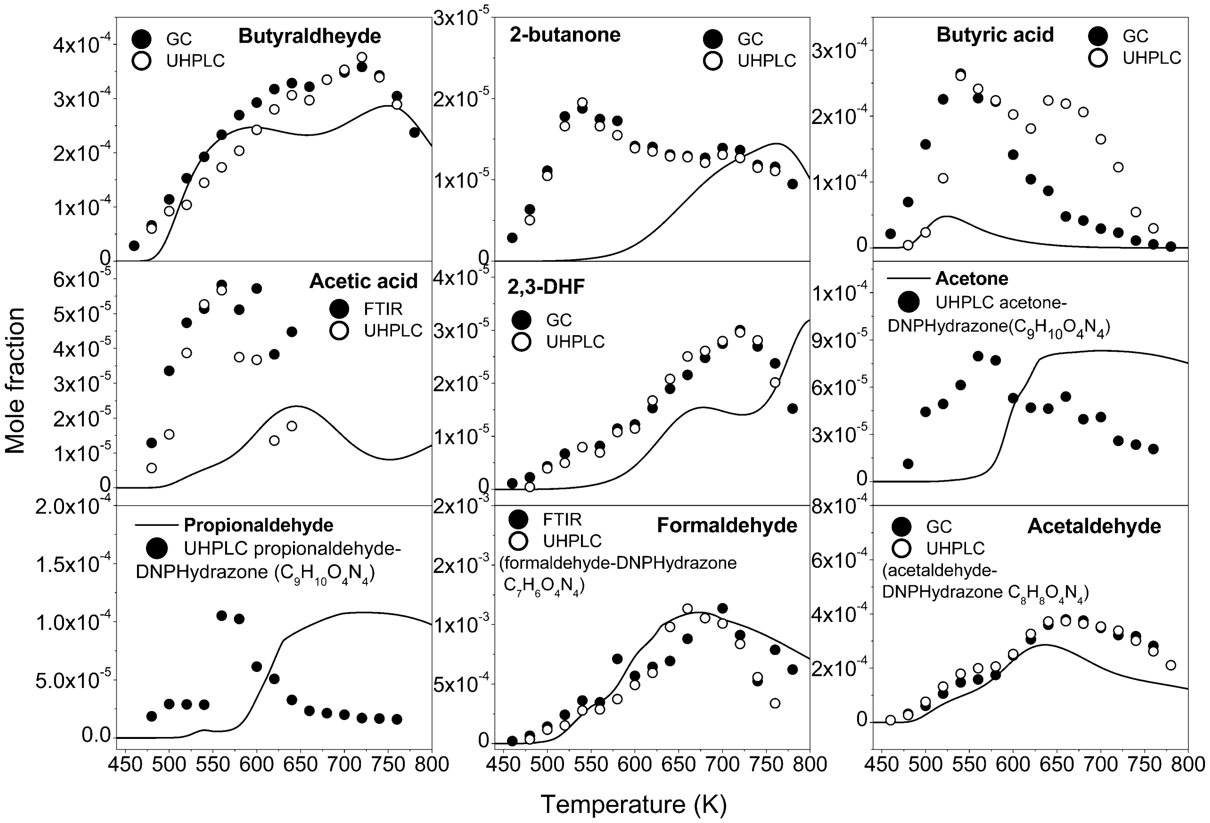

Figure S3. Mole fractions of aldehydes (formaldehyde, acetaldehyde, propionaldehyde, butyraldehyde), ketones (acetone, 2-butanone), carboxylic acids (acetic acid, butyric acid), and 2,3-DHF formed during the low temperature oxidation of 2500 ppm of di-n-butyl ether in a JSR. Lines represent the computed mole fractions, symbols represent GC, FTIR, and UHPLC-HRMS experimental results. UHPLC qualitative data were scaled to GC or FTIR quantitative data.

N.B: Propionaldehyde and acetone were co-eluted in GC and LC analyses. Thus, experimental results are presented for their DNPHydrazone derivatives ($C_9H_{10}O_4N_4$) which could be separated by C_{18} UHPLC-HRMS (retention times = 14.52 and 14.92 min, respectively). For formaldehyde and acetaldehyde (m/z < 50) experimental results were also obtained with their DNPHydrazone derivatives ($C_7H_6O_4N_4$, $C_8H_8O_4N_4$ respectively). Note that due to the high concentration of formaldehyde in the di-n-butyl ether oxidation samples, samples had to be diluted (1 µl of sample in 1 ml of ACN) before DNPH derivatization.

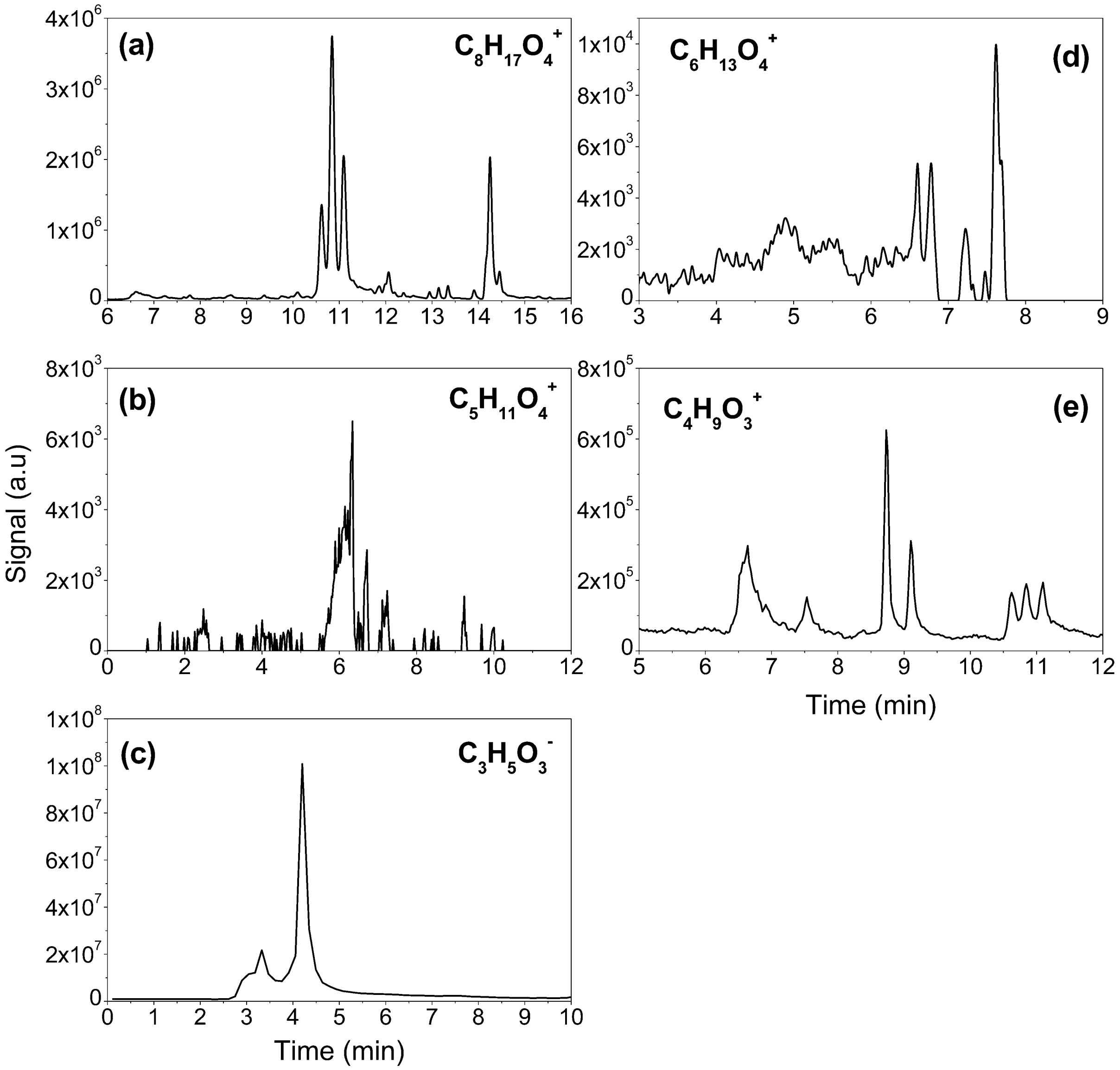

Figure S4. Chromatograms showing $C_{3,4,5,6,8}$ ketohydroperoxides formed during the oxidation of di-n-butyl ether in a JSR. (a) C_{18} UHPLC positive APCI HRMS signal of $C_8H_{16}O_4$ ($C_8H_{17}O_4^+$, with *m*/*z* 177.1121), (b) C_{18} UHPLC positive APCI HRMS signal of $C_5H_{10}O_4$ ($C_5H_{11}O_4^+$, with *m*/*z* 135.0651), (c) Silica HPLC negative APCI HRMS signal of $C_3H_6O_3$ ($C_3H_5O_3^-$, with *m*/*z* 89.0243, (d) C_{18} UHPLC positive APCI HRMS signal of $C_6H_{12}O_4$ ($C_6H_{13}O_4^+$, with *m*/*z* 149.0807), and (e) $C_4H_8O_3$ signal ($C_4H_9O_3^+$, with *m*/*z* 105.0543) with C_{18} UHPLC positive APCI HRMS.

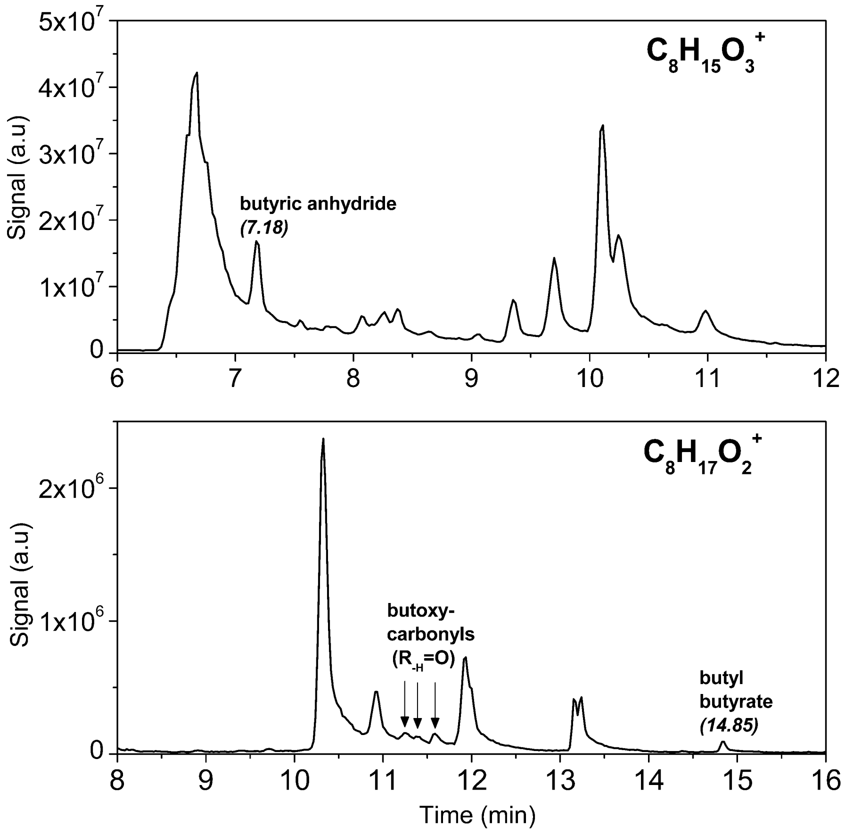

Figure S5. (Top) chromatogram showing the formation of $C_8H_{14}O_3$ diones during the oxidation of di-n-butyl ether. Butyric anhydride was identified at t=7.18 min. Analyses were performed using a C_{18} UHPLC column, positive APCI HRMS ($C_8H_{15}O_3^+$ with *m*/*z* 159.1015). (Bottom) chromatogram showing the formation of cyclic ethers, butyl butyrate, and butoxy-carbonyls ($C_8H_{16}O_2$) during the oxidation of di-n-butyl ether. Butyl butyrate was identified at t=14.85 min, and butoxy-carbonyls in the range 11.10 to 11.65 min. The other peaks correspond to cyclic ethers. Analyses were performed using a C_{18} UHPLC column and positive APCI HRMS ($C_8H_{17}O_2^+$, with *m*/*z* 145.1222).

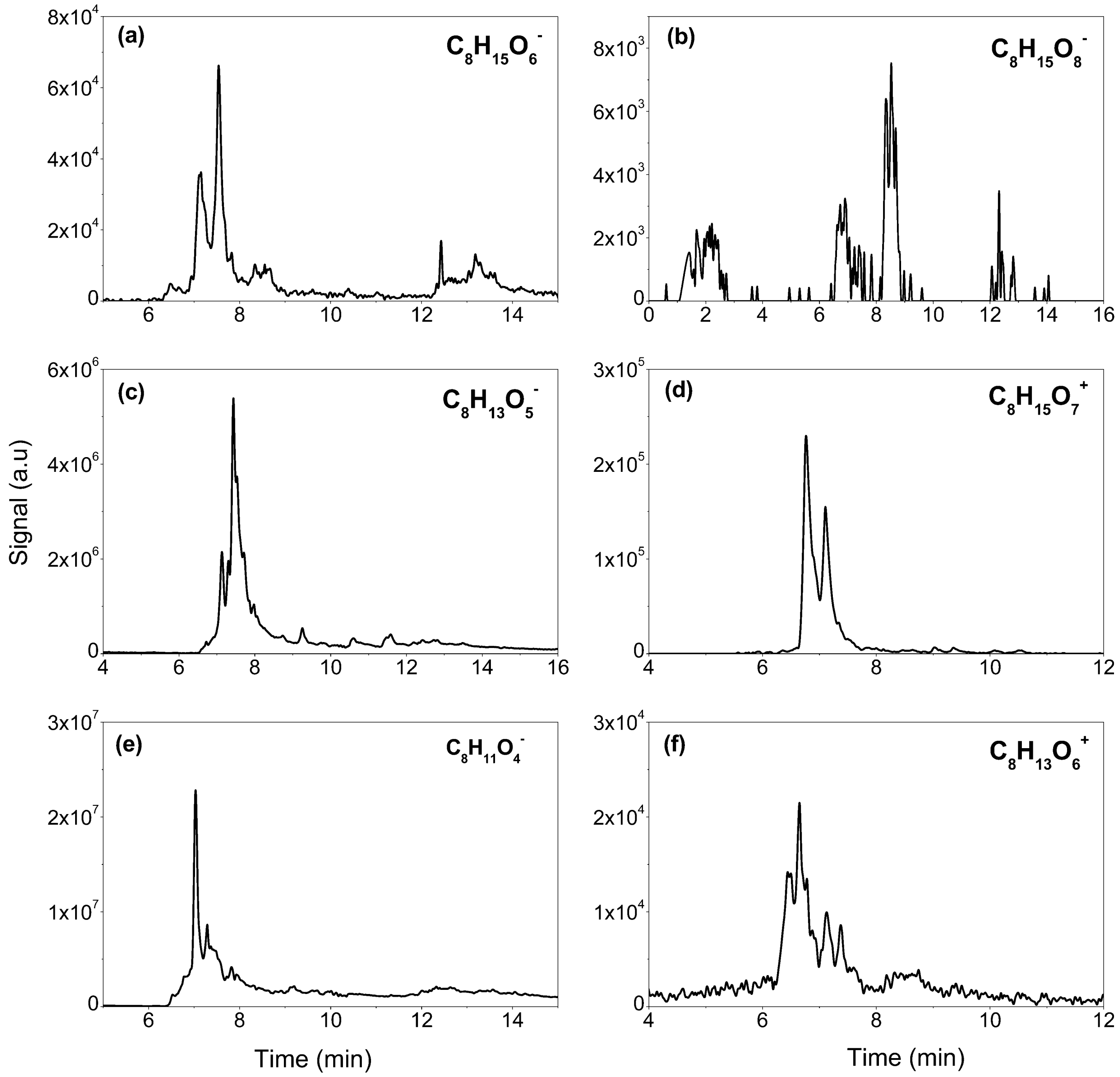

Figure S6. Chromatograms showing HOMs formed during the low temperature oxidation of 2500 ppm of di-n-butyl ether in a JSR. (a) $C_8H_{16}O_6$ (negative APCI, $C_8H_{15}O_6^-$, *m/z* 207.0873), (b) $C_8H_{16}O_8$ (negative APCI, $C_8H_{15}O_8^-$, *m/z* 239.0771), (c) $C_8H_{14}O_5$ (negative APCI, $C_8H_{13}O_5^-$, *m/z* 189.0768), (d) $C_8H_{14}O_7$ (positive APCI, $C_8H_{15}O_7^+$, *m/z* 223.0812), (e) $C_8H_{12}O_4$ (negative APCI, $C_8H_{11}O_4^-$, *m/z* 171.0662), and (f) $C_8H_{12}O_6$ (positive APCI, $C_8H_{13}O_6^+$, *m/z* 205.0706. All HOMs were analyzed using a C_{18} UHPLC column.

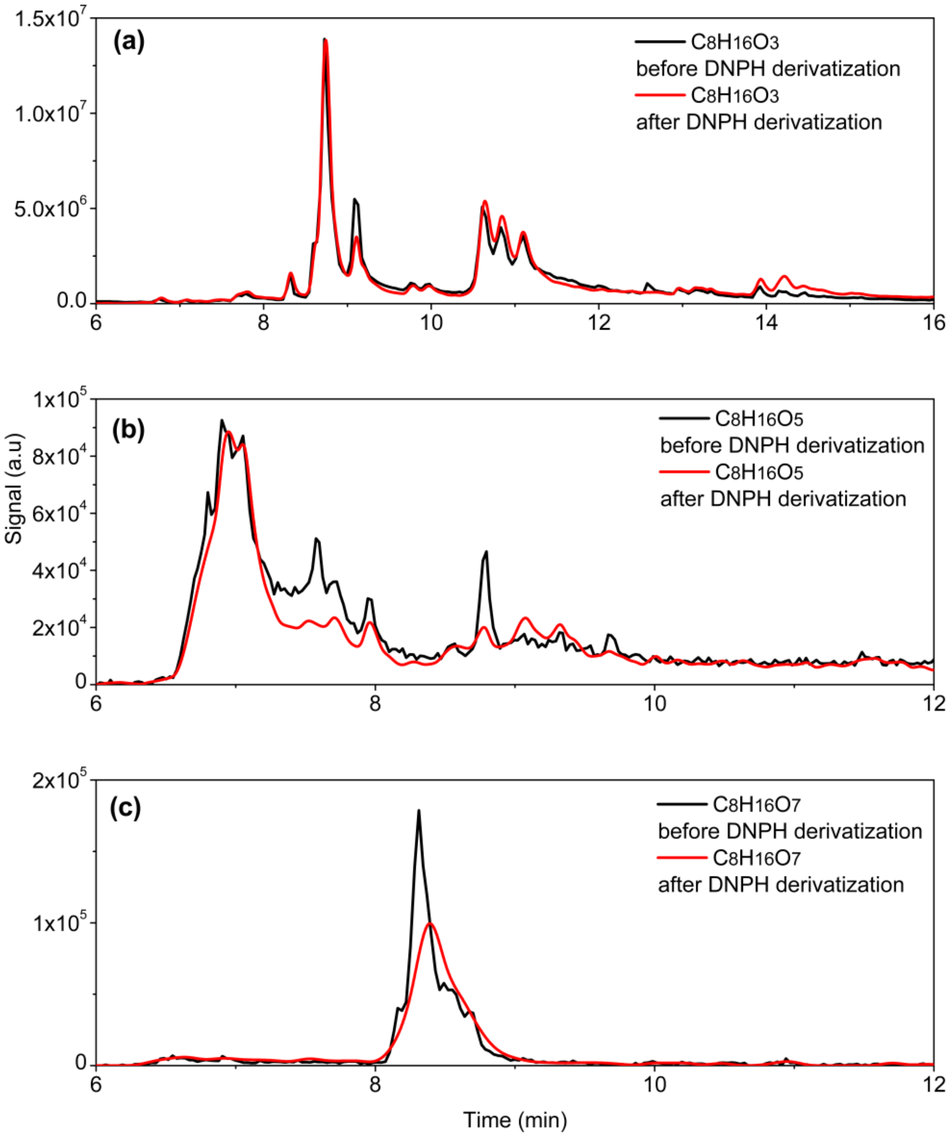

Figure S7. Chromatograms for $C_8H_{16}O_{3,5,7}$ obtained before and after 2,4-DNPH derivatization. A C_{18} UHPLC was used, positive and negative APCI HRMS analyses were performed. Peaks which decreasing intensities represent $C_8H_{16}O_{3,5,7}$ with a carbonyl function. They could be produced via an 'atmospheric oxidation route'. Peaks showing no reduced intensity after addition of 2,4-DNPH correspond to $C_8H_{16}O_{3,5,7}$ with no carbonyl function. They are expected to be unsaturated hydroperoxides formed via a 'combustion route'.

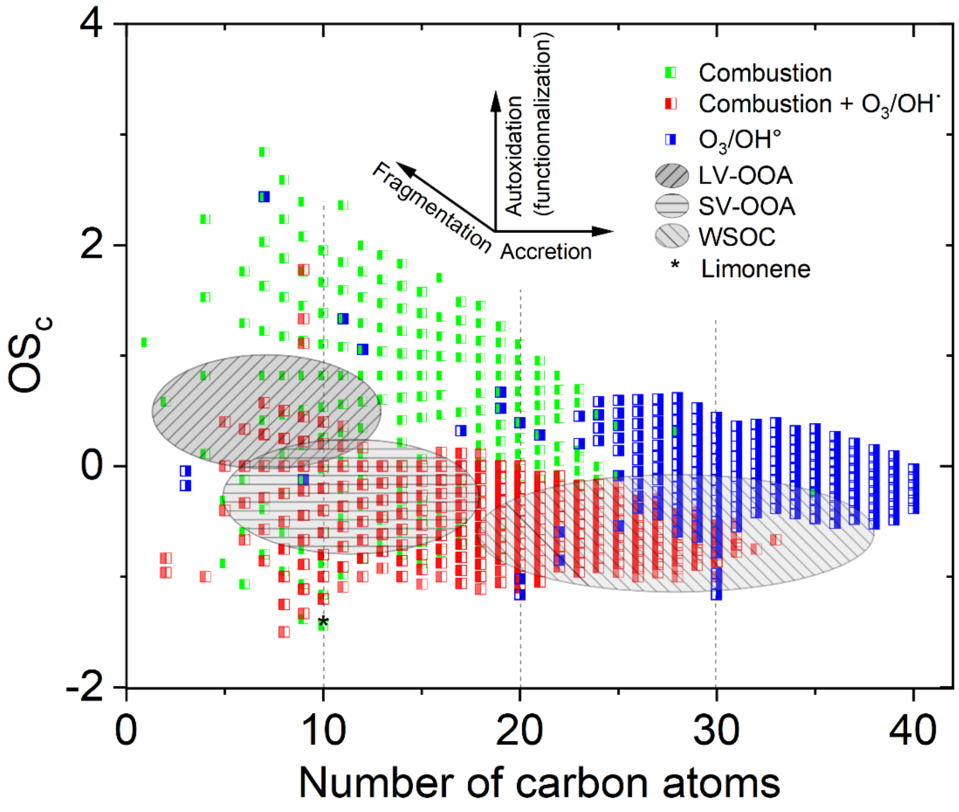

Figure S8. Variation of the OSc as a function of the number of carbon atoms in molecular formulae detected by negative FIA-HRMS of a limonene oxidation sample collected at 590 K and 1 atm.

Table S1. Conditions for GC, normal- and reverse-phase liquid chromatography, FTIR analyzes.

Analytical parameters	GC	LC	FIA
**Column**	(a) Coupling of two capillary columns: CP-Sil 5CB (1.2 µm, 0.32 mm, 25m) and DB-1 (3 µm, 0.53 mm, L=30m).<br>(b) CP-AL_2O_3/KCl (0.32 mm, 5µm, L=50m)<br>(c) CP-Carboplot (0.53mm, 50µm, L:25m)	(a) C_{18} (1.6 µm, 100Å,2.1mm, 100mm)<br>(b) Silica (5 µm, 250 × 2.1 mm)	–<br>–<br>–
**Mobile or gas phase**	(a) Helium, flow rate 0.7 ml/min.<br>(b) Helium, flow rate 1.5 ml/min.<br>(c) Nitrogen, flow rate 15 mL/min.	(§) H_2O+ACN (5 to 100% ACN, 250 µl/min during 20min)<br>(*) 0 to 5 min (constant 20% ACN+80%H_2O, 5 to 15 min: gradient 20 to 100% of ACN), 15 to 20min: 100%ACN, flow rate 350µl/min during 20 min.<br>(#) 95% ACN+ 5%H_2O, 250 µl/min during 15min	Flow rate 5 µl/min
**Temperature (°C)**	(a) constant at 35°C for 10 min, increasing by 4°C/min until 110°C, increase by 10°C/min until 200°C. Duration analysis: 39.75min<br>(b) T_0=70 °C, DT= 10°/min, T_f=200°C, keep 200°C for 10 min<br>(b) T_0=50°C, keep 50°C for 5 min	(a) and (b): 40	–<br>–<br>–
**Detector**	FID, q-MS<br>TCD	Orbitrap HRMS	Orbitrap HRMS
**Ionization**	EI (electronic impact 70 eV)	APCI (+/-) Sheath gas: 50 a.u<br>Auxiliary gas: 0 a.u<br>Capillary temperature: 320 °C<br>Vaporizer temperature: 80 °C	APCI (-)<br>Sheath gas: 10<br>Auxiliary gas: 1<br>Capillary temperature: 200 °C<br>Vaporizer: 120 °C

FTIR

FTIR analyses were performed under the following conditions: gas cell with a pathlength of 10m, pressure of 200 mbar and controlled temperature of 145°C.

(§): DBE-yl-hydroperoxides, unsaturated DBE-yl-hydroperoxides, $C_{4,5,6,8}$ ketohydroperoxides, cyclic ethers, diones, HOMs ($C_8H_{12}O_{4,6}$, $C_8H_{14}O_{3,5,7}$, and $C_8H_{16}O_{6,8}$)
(*): ROOR′: $C_{16}H_{34}O_4$, $C_{11}H_{24}O_3$, $C_{11}H_{22}O_3$, and $C_{10}H_{22}O_3$
(#): low mass and polar species: $C_3H_6O_3$ ketohydroperoxides and acetic acid.
